# Structure is more important than physiology for estimating intracanopy distributions of leaf temperatures

**DOI:** 10.1002/ece3.4046

**Published:** 2018-04-27

**Authors:** H. Arthur Woods, Marc Saudreau, Sylvain Pincebourde

**Affiliations:** ^1^ Division of Biological Sciences University of Montana Missoula MT USA; ^2^ INRA, UCA, UMR PIAF Clermont‐Ferrand France; ^3^ Institut de Recherche sur la Biologie de l'Insecte (IRBI) CNRS UMR 7261 Faculté des Sciences et Techniques Université François Rabelais Tours France

**Keywords:** behavioral thermoregulation, boundary layer, climate, climate change, insect, Jarvis parameters, leaf, leaf area density, leaf inclination angle distribution, numerical model, stomatal conductance, temperature, voxel

## Abstract

Estimating leaf temperature distributions (LTDs) in canopies is crucial in forest ecology. Leaf temperature affects the exchange of heat, water, and gases, and it alters the performance of leaf‐dwelling species such as arthropods, including pests and invaders. LTDs provide spatial variation that may allow arthropods to thermoregulate in the face of long‐term changes in mean temperature or incidence of extreme temperatures. Yet, recording LTDs for entire canopies remains challenging. Here, we use an energy‐exchange model (RATP) to examine the relative roles of climatic, structural, and physiological factors in influencing three‐dimensional LTDs in tree canopies. A Morris sensitivity analysis of 13 parameters showed, not surprisingly, that climatic factors had the greatest overall effect on LTDs. In addition, however, structural parameters had greater effects on LTDs than did leaf physiological parameters. Our results suggest that it is possible to infer forest canopy LTDs from the LTDs measured or simulated just at the surface of the canopy cover over a reasonable range of parameter values. This conclusion suggests that remote sensing data can be used to estimate 3D patterns of temperature variation from 2D images of vegetation surface temperatures. *Synthesis and applications*. Estimating the effects of LTDs on natural plant–insect communities will require extending canopy models beyond their current focus on individual species or crops. These models, however, contain many parameters, and applying the models to new species or to mixed natural canopies depends on identifying the parameters that matter most. Our results suggest that canopy structural parameters are more important determinants of LTDs than are the physiological parameters that tend to receive the most empirical attention.

## 1 INTRODUCTION

Estimating leaf temperature distributions (LTD) in canopies is fundamental in forest ecology (Jones, 1992; Nobel, 1999). Leaf temperature alters rates of photosynthesis, transpiration, and respiration (Collatz, Ball, Grivet, & Berry, 1991; Kobza & Edwards, 1987), with direct consequences for plant growth rate, energy and water status, and associated consequences for the local environment including soil moisture content and levels of atmospheric water vapor (Michaletz et al., 2016; Sellers, 1997). Temperature also alters the performance of species living in or on leaves, including rates of population growth of phytopathogens (Bernard, Sache, Suffert, & Chelle, 2013; Chelle, 2005) and of feeding, growth, and survival by insect herbivores (Bale et al., 2002; Kingsolver, 2009; Pincebourde & Woods, 2012).

Canopy temperature distributions can be measured using several methods, including temperature‐sensing probes such as thermocouples (Linacre, 1967), infrared thermography (Faye, Rebaudo, Yánez‐Cajo, Cauvy‐Fraunié, & Dangles, 2016; Leuzinger & Körner, 2007; Pincebourde & Suppo, 2016; Scherrer, Bader, & Körner, 2011), and isotopic analyses (Helliker & Richter, 2008). These studies generally have reported high variation in leaf temperatures between species (Michaletz et al., 2016 but see Helliker & Richter, 2008), and a few also illustrate *within*‐canopy thermal heterogeneity (Leuzinger & Körner, 2007; Scherrer et al., 2011). However, all of these approaches have drawbacks that prevent them from revealing the full extent of insect‐relevant variation in temperature within canopies: It is difficult to get extensive spatial coverage with thermocouples, IR thermography provides 2D views (top or side) of 3D canopies, and isotopic analyses integrate leaf conditions over large spatial and temporal scales. For estimating effects on canopy insects, we need better estimates of thermal diversity (i.e., LTDs) *within* individual canopies, which sets the local bounds of temperatures available to individual insect herbivores and to their local populations.

Besides mean temperature, which is of primary interest in many contexts, other aspects of variation in temperature come into play when local populations are exposed to environmental change. For example, changes in the spatial (or temporal) variance in temperature can mask or reverse the effect of a change in mean temperature (Benedetti‐Cecchi, Bertocci, Vaselli, & Maggi, 2006; Dillon et al., 2016; Vasseur et al., 2014). In addition, temperature variance at local scales defines the range available for organisms (Faye et al., 2016; Pincebourde & Suppo, 2016; Woods, Dillon, & Pincebourde, 2015) to use during behavioral thermoregulation, which is a key process by which ectotherms achieve high rates of performance (Woods et al., 2015) and avoid temperature extremes (Kearney, Shine, & Porter, 2009; Sunday et al., 2014). Finally, patterns of spatial autocorrelation in temperature matter for mobile organisms (Sears et al., 2016). In particular, ectotherms may pay lower energetic costs when favorable temperatures are less aggregated (i.e., are more patchily distributed) in space (Faye, Rebaudo, Carpio, Herrera, & Dangles, 2017; Sears et al., 2016). Several indices of aggregation have been developed by landscape scientists but have been used only rarely by ecologists working at local (Faye et al. 2016, Faye et al., 2017: scale of a crop field) and small scales (Caillon, Suppo, Casas, Woods, & Pincebourde, 2014: scale of a single leaf).

Biophysical models have become common, powerful tools for analyzing the complex interactions among fluxes of heat, momentum, and mass that occur in plant canopies. Papers using these models, however, generally do not describe patterns of temperature *variation* that are relevant to insects, primarily because they are concerned with plant physiology: photosynthesis, transpiration, and respiration (e.g., Tuzet, Perrier, & Leuning, 2003; for exceptions, see Dai, Dickinson, & Wang, 2004; Bauerle, Bowden, Wang, & Shahba, 2009; Bailey, Stoll, Pardyjak, & Miller, 2016). In addition, most models do not provide adequate information about 3D patterns of temperature variation at spatial scales relevant to insects (on the order of cm to m) as they focus on whole canopies, and they generally take either a multilayer or a big‐leaf approach (Baldocchi, Wilson, & Gu, 2002; Bonan, 1996; Dai et al., 2004; Flerchinger, Reba, Link, & Marks, 2016; Leuning, Kelliher, de Pury, & Schulze, 1995; Pyles, Weare, & Pawu, 2000; Sellers, 1997; Wang & Jarvis, 1990; Wohlfahrt, 2004). Both multilayer and big‐leaf models, however, are effectively one‐dimensional models that describe changes along the vertical axis of a canopy rather than throughout full 3D canopies. Focusing on much smaller spatial scales, Saudreau et al. (2017) recently modeled how interactions between irradiance and leaf microtopography drive patterns of temperature variation within single leaves. Temperature variation at such small scales may also be exploited by individual insects (Caillon et al., 2014), but that spatial scale also does not capture the intracanopy scale that is relevant to most mobile insect herbivores.

The RATP model (Radiation Absorption, Transpiration, and Photosynthesis; Sinoquet, Le Roux, Adam, Ameglio, & Daudet, 2001) provides a good platform for bridging this spatial gap. This mechanistic biophysical model discretizes space (the unit cell is called a voxel) with a predefined dimension, allowing the user to adapt the spatial scale at which the model predicts leaf temperatures. The use of voxels makes it possible to apply the Beer–Lambert law of extinction of light across vegetation and to lower computational time considerably compared to a model simulating leaf‐scale processes (see Saudreau et al., 2017). This model has been used successfully to estimate LTDs in apple trees and several other cultivated species at a voxel size of 20 cm (Ngao, Adam, & Saudreau, 2017). In addition, it has been coupled to more detailed models of heat budgets of insect‐built structures (leaf mines) and of fruits in apple trees (Pincebourde, Sinoquet, Combes, & Casas, 2007). It has not yet been applied to trees in the wild or to forest stands, but doing so presents few conceptual problems.

The RATP model has a large number of parameters and explicitly uses environmental forcing variables that fall into three groups: (1) environmental—air temperature, wind speed, relative humidity, amounts of incoming short‐ and longwave irradiance, and the relative proportions of direct vs. diffuse irradiance; (2) structural—leaf area density and leaf inclination angle distribution; and (3) physiological—stomatal effects on evapotranspiration via stomatal sensitivity to temperature, water status, vapor pressure deficit, and light. It is not obvious a priori which parameters deserve the most attention. Clearly, environmental parameters strongly influence leaf temperature (Monteith & Unsworth, 2008; Nobel, 1999), and they are among the easiest to collect. However, even the relative ordering of those variables is unclear (e.g., is LTD influenced more by variation in air temperature or incident irradiation?). Many studies have considered the impact and relative importance of canopy structure on leaf functioning (Baldocchi et al., 2002; Caldwell, Meister, Tenhunen, & Lange, 1986). However, most of these focus on photosynthesis and transpiration (Niinemets, Keenan, & Hallik, 2015). Even though leaf temperature is an integral part of many of the models used, few authors have analyzed leaf temperature explicitly, including its distribution at the scale of a single canopy or continuous canopies. This is a key gap that prevents us from connecting leaf temperature distributions to other trophic levels.

Here, we present a Morris sensitivity analysis (Campolongo, Cariboni, & Saltelli, 2007; King & Perera, 2013; Morris, 1991) of how LTDs depend on climatic, structural, and physiological parameters in the RATP model. The Morris analysis subsamples combinations of parameters from throughout the full, multidimensional parameter space and then ranks individual parameters according to their weight on the output variable (Morris, 1991). We developed our analysis using the well‐described architecture and physiology of apple trees, *Malus domestica* (Saudreau et al., 2013). Apple is probably the plant species with the most detailed dataset on its architecture (Saudreau et al., 2017; Sinoquet et al., 2009), although quite complex methodologies have been developed to capture the architecture of forest trees (e.g., Lintunen, Sievänen, Kaitaniemi, & Perttunen, 2011). We also used a common voxel size (20 cm), which was shown to predict well the leaf temperatures at local and whole canopy scales. We first apply the Morris analysis to the canopy of a single isolated tree and then to a virtual stand composed of several trees with contiguous canopies. Finally, we leverage the RATP model to provide 2D views of the 3D distribution of leaf temperatures in the canopies. These 2D views approximate how infrared devices image canopies from a distance (seeing only leaves on, e.g., the top or side). The comparison of LTDs from 2D and 3D views quantifies the error in LTDs when using remote sensing.

## 2 METHODS

### 2.1 RATP model

The RATP model was designed to simulate the spatial distribution of radiation and leaf‐gas exchanges within a plant canopy as a function of its geometry, the surrounding climate, and the physical and physiological properties of leaves (Sinoquet et al., 2001). The RATP model is based on many equations and requires many inputs: 10 above‐canopy forcing climatic variables, more than 20 parameters related to the optical and physiological properties of the leaves, and five input parameters describing the spatial distribution of leaves within the canopy. The main model features are summarized below, and a fuller description of the model and equations can be found in Sinoquet et al. (2001) and in Appendix S1. In the RATP model, a turbid medium approach is used to compute radiation transfer in a canopy composed of one or several species. Briefly, the model accounts for down‐welling shortwave radiation from the sun (direct and diffuse) in PAR and NIR wavebands, atmospheric longwave radiation (sky irradiance), longwave radiation from the soil, and longwave exchange among leaves. Whenever possible, down‐welling fluxes are set with measured values. If these are not available, they are estimated from empirical relationships based on classical meteorological data. For instance, the partition of the sun irradiance between direct and diffuse parts can be inferred from the clear sky index (Reindl, Beckman, & Duffie, 1990), and the sky longwave irradiance from air temperature and vapor pressure of water in the air (Iziomon, Mayer, & Matzarakis, 2003). The plant canopy is embedded in an array of 3D voxels (set to the optimal dimensions of 20 × 20 × 20 cm; Sinoquet, Sonohat, Phattaralerphong, & Godin, 2005), and the canopy structure is entirely defined by the spatial distribution of the leaf surface area and the inclination angles of the leaves. Each individual voxel is characterized by a leaf area density and composed of a sunlit leaf surface area that intercepts both direct and diffuse radiation and a shaded leaf surface area that intercepts diffuse radiation only. For simplicity, we considered canopies composed of leaves only, and all other organs such as branches and fruits were neglected. To estimate local leaf temperature, the energy balance equation between incoming and outgoing fluxes is closed in each voxel for sunlit and shaded leaves.

Achieving energy balance requires additional information about sensible and latent heat fluxes (convective transfer between leaf and air, and transpiration cooling, respectively). In the RATP model, sensible heat flux lost or gained by a leaf is assumed to be related to a boundary layer conductance and the difference in temperature between the leaf and the surrounding air (Monteith & Unsworth, 2008). The boundary layer conductance is linearly related to the local wind velocity (Daudet, Le Roux, Sinoquet, & Adam, 1999). We accounted for wind attenuation in the canopy using an empirical relationship between relative wind speed and the cumulative leaf area along the wind path (Daudet et al., 1999). The transpiration rate of a leaf is driven by the physiological response of the plant (stomatal conductance) and the evaporative demand (Monteith & Unsworth, 2008). Stomatal conductance is controlled by the microclimate at the leaf surface (PAR intercepted, CO_2_ and VPD), the leaf temperature, and the leaf physiological state following the Jarvis model (Jarvis, 1976) assuming no interactions between these variables on the stomatal response. The overall leaf conductance for water vapor transfer is computed by combining boundary layer and stomatal conductance of both upper and lower leaf surfaces. The overall set of equations in the RATP model consists of a nonlinear system with unknown variables, the temperature of sunlit or shaded leaf areas, to be solved (Appendix S1). Brent's iterative method (Brent, 1974) is used to solve the model. Model outputs of concern are the irradiance at the leaf surface and temperature of the shaded and sunlit area of foliage at the voxel scale.

In all subsequent analyses, we analyzed patterns of thermal variation in the entire 3D canopy of the forest stand (results for single tree are presented in Supporting Information) and just in the 2D views from above. By comparing 2D and 3D patterns of variation, we were able to estimate the utility of flyover views (e.g., what an infrared‐equipped UAV would see) for estimating spatial patterns of thermal variation throughout the entire 3D canopy.

### 2.2 Parameter values

**Figure 1:**
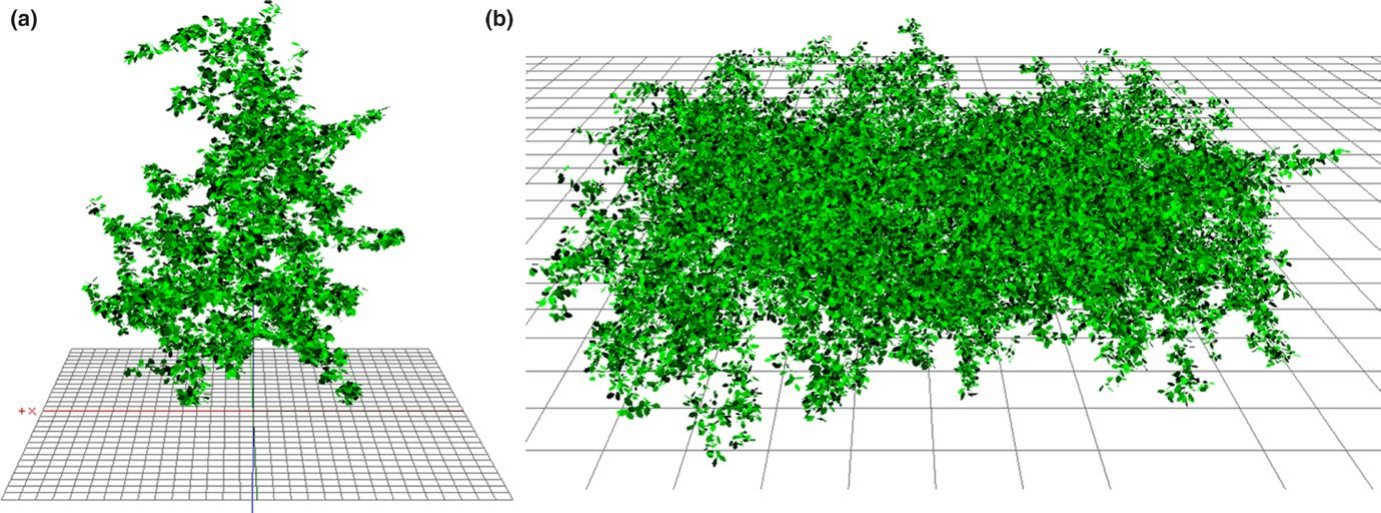
Three‐dimensional view of the isolated apple tree (a) and the virtual stand (b) used in the simulations. The virtual stand was made artificially by putting together twenty isolated tree canopies to construct a continuous canopy. The total leaf surface area was 24.3 m^2^ (with 10,214 leaves) and 202 m^2^ (with 93,992 leaves) for the isolated tree and the continuous canopy, respectively. Space was discretized into 574 and 5,340 voxels (20 × 20 × 20 cm) for the isolated tree and the continuous canopy, respectively. A total of 107 and 623 voxels can be seen from the top of the isolated tree and the continuous canopy, respectively

Because the model has been applied most extensively to apple trees, we used as the base set of parameters those that have been measured for apples in France. Briefly, a set of apple trees were digitized leaf by leaf, reporting the 3D coordinates of each individual leaf and its three Euler angles (Figure 1; Pincebourde et al., 2007; Sinoquet et al., 2009). From these digitized trees, two different tree plots were constructed: a first one consisting of a single tree (Figure 1a) and a second one composed of 20 apple trees in a forest stand (Figure 1b) in which the model's bounding box cut off the outermost sections. In the stand, trees were regularly spaced on a grid of 125 cm by 150 cm. These architectural traits directly defined the LAD and LIAD parameters (Table 1). In addition, physiological parameters related to stomatal responses (Jarvis functions) were measured on these same trees (Table 1; Pincebourde et al., 2007; Saudreau et al., 2013). For the parameters in the Morris sensitivity analysis (Table 1), we took ranges of values that are typical for temperate regions where apple trees are cultivated in France.

**Table 1:** Parameters and the range of each used in simulations. Some parameters are dimensionless

Parameter/Variable	Definition	Bounds and units
*T*_air_	Air temperature	[10 — 40]°C
RH	Relative humidity	[20 — 100] %
Irradiance	Total irradiation	[200 — 1,200] W/m^2^
Wind	Wind speed	[0 — 5] m/s
Kt	Hourly diffuse to direct ratio (Reindl et al., 1990) $R_{\mathrm{diff}}/R_{\mathrm{global}}\;=\;\left\{\begin{array}{c} 1.0-0.17\;\text{Kt}\;\text{for}\;\text{Kt}\;\leq \;0.3\\ 1.45-1.67\;\text{Kt}\;\;\;\text{for}\;0.3\;<\;\text{Kt}\;<\;0.78\\ 0.147\;\;\;\text{for}\;\text{Kt}\;\geq \;0.78 \end{array}\right.$	[0 — 1]
LAD	LAD = a*LAD_init_	[0.5 — 2]
LIAD	LIAD distribution (Wang, Li, & Su, 2007)	Defined functions: Planophile, Erectophile, Plagiophile, Uniform
*b*_temp_	Stomatal response to leaf temperature $f_{1}\left(T_{\mathrm{Leaf}}\right)\;=\;\frac{0.97498}{1\;+\;{\left(\frac{T_{\mathrm{Leaf}}-b_{\mathrm{temp}}}{c_{\mathrm{temp}}}\right)}^{2}}$	[10 — 35]°C
*c*_temp_	Stomatal response to leaf temperature $f_{1}\left(T_{\mathrm{Leaf}}\right)\;=\;\frac{0.97498}{1\;+\;{\left(\frac{T_{\mathrm{Leaf}}-b_{\mathrm{temp}}}{c_{\mathrm{temp}}}\right)}^{2}}$	[5 — 15]°C
Gsmax	Maximal stomatal conductance	[1e‐3 — 4e‐3] m/s
*d*_PAR_	Stomatal response to PAR $f_{3}\left(\text{PAR}\right)\;=\;\frac{a_{\mathrm{PAR}}\text{PAR}\;+\;b_{\mathrm{PAR}}}{\text{PAR}\;+\;d_{\mathrm{PAR}}}$ With *b*_PAR_ = *d*_PAR_/10 and *a*_PAR_ = (2,000 + *d*_PAR_ − *b*_PAR_)/2,000	[100 — 5,000] μmol m^−2^ s^−1^
*a*_VPD_	Stomatal response to VPD $f_{2}\left(\text{VPD}\right)\;=\;\left\{\begin{array}{c} 1\;\text{if VPD}\;<\;a_{\mathrm{VPD}}\\ b_{\mathrm{VPD}}\;+\;c_{\mathrm{VPD}}\text{VPD if VPD}\;>\;a_{\mathrm{VPD}} \end{array}\right.$( )with *f*_2_(VPD ≥ VPD_max_) = 0, *b*_VPD_ = 1‐*c*_VPD_*a_VPD_ and *c*_VPD_ = 1/(*a*_VPD_ − VPD_max_)	[200 — 2,000] Pa
VPD_max_	Stomatal response to VPD $f_{2}\left(\text{VPD}\right)\;=\;\left\{\begin{array}{c} 1\;\text{if VPD}\;<\;a_{\mathrm{VPD}}\\ b\;+\;c\text{VPD}\;\text{if VPD}\;>\;a_{\mathrm{VPD}} \end{array}\right.$ with *f*_2_(VPD ≥ VPD_max_) = 0, *b*_VPD_ = 1 − *c*_VPD_*a_VPD_ and $c_{\mathrm{VPD}}\;=\;1/\left(a_{\mathrm{VPD}}-{\text{VPD}}_{\max}\right)$	[3,000 — 5,000] Pa

During the simulations, optical and physiological parameters other than those used in the Morris analysis (Table 1), including the spatial arrangement of 3D voxels, were kept constant. The 3D space was discretized into voxels of 20 × 20 × 20 cm size, leading to 574 and 5,340 voxels for the isolated tree and the forest stand, respectively. The simulations were performed for a given day (day of year 234), time (12 h GMT), and location on the earth's surface (France: 45° north latitude, and 0° east longitude). The sun was thus positioned in the sky at an elevation angle of 68° and an azimuthal angle from north of −178°.

### 2.3 Statistical summaries

The statistical analysis focused primarily on the sunlit portion of the foliage, which contained most of the temperature variation, but included some analysis of shaded portions too. Shaded portions were always close to ambient air temperature. For each simulation run, several key statistical parameters were computed. Heterogeneity can be described in terms of composition and configuration. Composition was described by the mean and variance of temperature at the canopy scale. Configuration was quantified using two spatial correlation indices: Moran's I (I_Moran_) and Geary's C (C_Geary_) indexes; results for Moran's I are presented in the main text and for Geary's C in Appendices S2 and S3. These indices reflect the extent to which patches (voxels) with similar temperatures are aggregated in space (Jumars, Thistle, & Jones, 1977), here at the scale of the canopy. Moran's I is based on a crossed‐product centered to the overall mean, while Geary's C is sensitive to the deviation between pairs of points independent of the mean (Fortin, Drapeau, & Legendre, 1989; Jumars et al., 1977). I_Moran_ is zero when the spatial distribution is random, and it moves toward −1 and +1 for dispersed and clustered distribution, respectively. C_Geary_ departs from 1 (no spatial autocorrelation) down to zero or above 1 for increasingly negative or positive spatial autocorrelation, respectively. Briefly, Moran's I detects aggregation due to extreme temperature values in several adjacent voxels, whereas Geary's C tests whether adjacent voxels contain similar temperatures (Jumars et al., 1977). Moran's I is often seen as a global, and Geary's C as a local, index of aggregation.

### 2.4 Sensitivity analyses

The Morris approach (Morris, 1991) provides an efficient way of exploring large parameter spaces. In our case, we used 13 parameters and variables with four levels each, or 4^13^ = 67,108,864 unique combinations of values. Because simulating each set takes several minutes on our computers, exploring the total factorial space is effectively impossible. The Morris approach cuts down on total coverage by taking a kind of random but well‐distributed walk through the 13‐dimensional hypercube of parameter values. In the analyses presented below, for example, we explored only 5,600 unique parameter combinations—which is <0.01% of the total number—but were still able to identify the relative influence of individual parameters on simulation outcomes. For each of the *i* parameters (among environmental, structural, and physiological groups), the analysis calculates two parameters, $\mu_{i}^{*}$ and σ_*i*_, which refer, respectively, to the overall strength of the effect of the *i*th parameter on simulation output values and the strength of its interactions with other parameters (King & Perera, 2013). This analysis was applied to the different statistical outputs explained above: mean leaf temperature, canopy scale variance in leaf temperature, and the Moran and Geary indices.

Once the relative importance of parameters was established using the Morris approach, we used standard sensitivity analyses to visualize the quantitative effect of four of those parameters—wind speed, irradiance, LAD, and Gsmax, spanning the range of values of μ*—on the mean and variance of temperature within canopies. These standard sensitivity analyses were performed by establishing a common set of parameter values and then varying each of the four focal parameters individually across its range of possible values (see Table 1).

## 3 RESULTS

### 3.1 RATP model outputs

**Figure 2:**
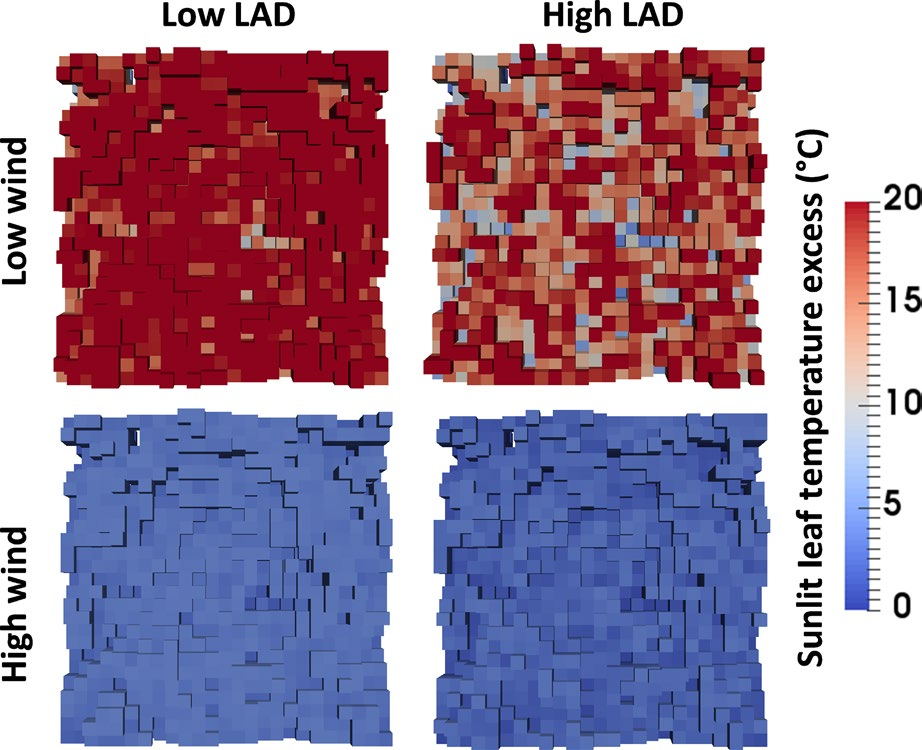
Two‐dimensional presentation of the spatial distribution of sunlit leaf temperature excess (i.e., leaf temperature minus air temperature) for top view voxels in a portion of the continuous canopy (i.e., when the canopy is viewed from above). In this example, four different simulations are shown, under low and high wind speed (0 and 5 m/s) factored with low and high leaf area density (LAD, 2.36 and 9.46 m^2^/m^3^). Colors refer to the amplitude of the sunlit leaf temperature excess. The voxels appearing in white correspond to gaps in the canopy, through which the ground can be seen from above the canopy. In all simulations, the other parameters were fixed: irradiance = 1,200 W/m^2^; air temperature = 20 °C; Kt = 0; Gsmax = 0.004 m/s

For a given set of parameters, the RATP model calculates steady‐state temperatures of the sunlit and shaded portions of each voxel across the entire 3D canopy. We analyzed the full 3D distributions of voxel temperatures and also the subset at the top of the canopy that one would see during a flyover (Figure 2). Figure 2 illustrates the large impact of LAD on the spatial configuration of the leaf temperature distribution (i.e., less aggregated and more dispersed at high LAD) and the large influence of wind speed, which homogenizes and cool down canopy leaf temperatures.

### 3.2 Ranking the relative importance of parameters

**Figure 3:**
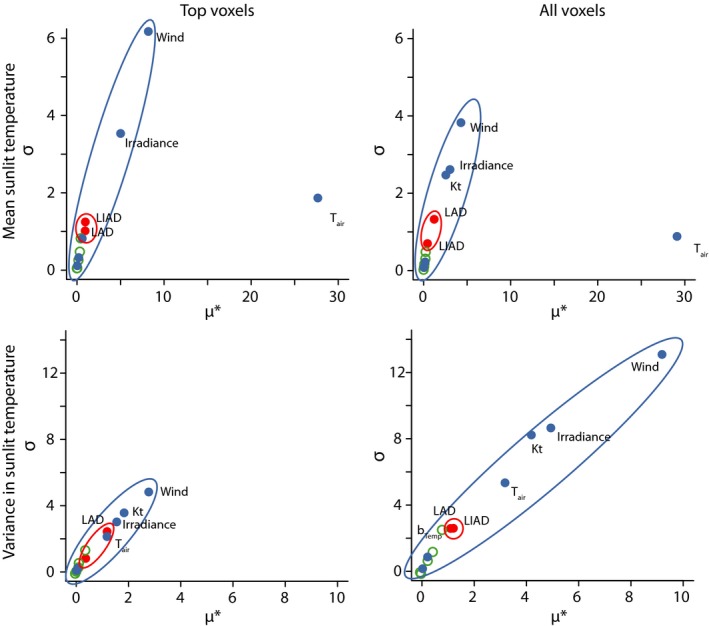
Morris analysis of the continuous canopy for all top‐viewed sunlit voxels (two‐dimensional, left) and all voxels (three‐dimensional, right) for mean temperature (top) and variance in temperature (bottom). Parameter types are color‐coded: Blue indicates climatic parameters, red structural, and green physiological. Ellipses were drawn for clarity. Clearly, climatic parameters have strong effects on both mean and variance of canopy voxels. Of the remaining two classes, however, structural parameters (LAD and LIAD) had, in almost all cases, stronger effects on mean and variance of voxel temperatures

The Morris analysis ranked the 13 parameters according to their μ* (overall relative impact of each parameter) and σ (the extent to which a given parameter interacts with others). Overall, the analysis indicated that the physiological parameters affected LTDs much less than did structural parameters and that the climatic parameters were the most influential (Figure 3).

Mean leaf temperature was driven mainly by climatic parameters, and in particular air temperature (highest μ*) and wind speed (highest σ) (Figure 3). These two parameters affected mean leaf temperature similarly for top‐viewed and all voxels (Figure 3a,b). The variance in leaf temperature was driven mostly by wind speed and to a lesser extent by the other climatic parameters, except humidity (Figure 3). However, the structural parameter LAD was also important, especially for the thermal variance in the top‐viewed voxel (Figure 3c,d). In general, we observed a positive relationship between the impact (μ*) and the interaction strength (σ) of parameters, except for air temperature, which had a high impact on mean leaf temperature but interacted little with other parameters.

**Figure 4:**
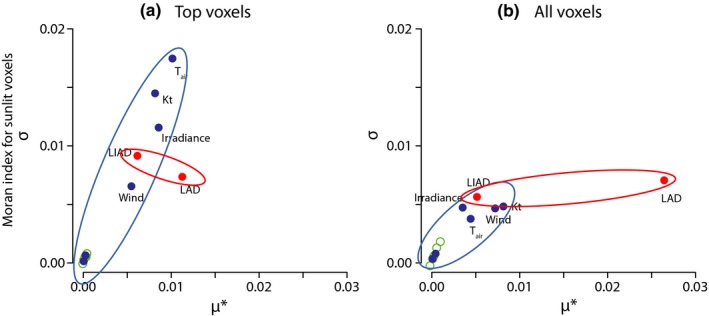
Morris analysis of the Moran index (an index of spatial aggregation of voxel temperatures) for (a) top‐viewed voxels (two‐dimensional, left) and (b) all voxels (three‐dimensional, right) in the continuous canopy. Parameter types are color‐coded: Blue indicates climatic parameters, red structural, and green physiological. Ellipses were drawn for clarity. Both climatic and structural parameters have strong effects on spatial aggregation of temperatures. The effects of LAD are especially pronounced and are magnified in Figure 4b because of strong vertical differences in the canopy between top and interior voxels

The Morris analysis showed further that the spatial autocorrelation of leaf temperatures (I_Moran_ and C_Geary_) was affected primarily by the structural parameter LAD and by climatic variables such as air temperature, irradiance, and Kt (Figure 4). The low σ of LAD for the Moran index indicates that this structural parameter does not interact strongly with the other parameters. Again, the physiological parameters were not important for the spatial configuration of LTDs for all and top voxels (Figure 4).

### 3.3 Standard sensitivity analysis

**Figure 5:**
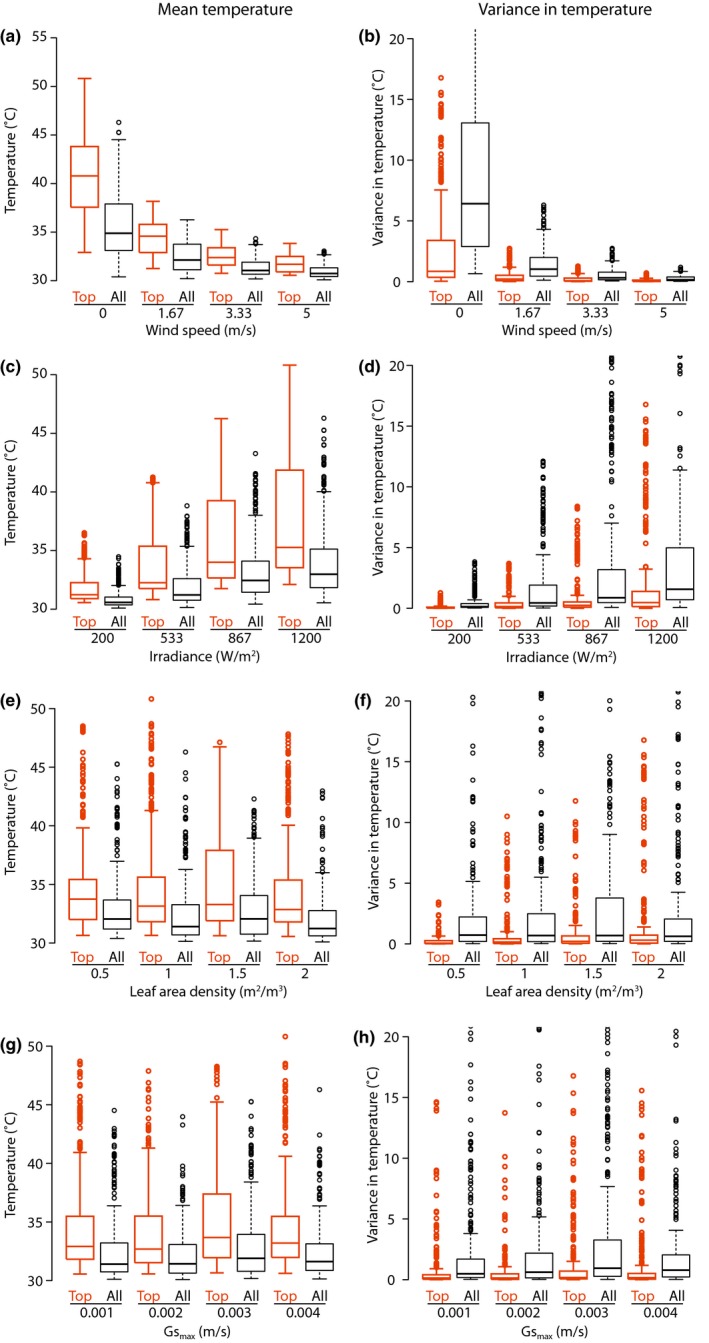
Magnitude of effects on mean and variance of temperature (in a continuous canopy) of sunlit portions of voxels of changing individual parameters with different values of Morris μ* (Appendix S2). For this illustration, we used only simulations in which Tair = 30°C (approx. ¼ of the entire set of 5,600 simulations). In each panel, we show boxplots of mean canopy temperature (each boxplot contains ¼ of the subset, or about 350, of the simulations) as a function of varying the focal parameter over its four levels used in the Morris analysis. In addition, for each simulation output, we calculate the means of all (sunlit portions of) voxels in the canopy and for just upward‐looking voxels. Effects of wind speed on mean (a) and variance (b) of canopy temperature. (c, d) Effects of irradiance. (e, f) Effects of LAD. (g, h) Effects of Gsmax

The LTDs for top and all voxels were compared by visually inspecting the quantitative difference between the respective box plots obtained by varying wind speed and irradiance, two of the most important parameters from the Morris analysis above, and the LAD, an intermediate parameter, and finally Gsmax, which is not expected to generate significant variations among voxels according to the Morris analysis (Figure 5). Overall, the mean leaf temperature distributions of top and all voxels were close to each other over a wide range of parameter values, except under low wind speed (the median of the LTD for top voxels was about 5°C higher than for all voxels) and high irradiance (the 50% central values of the LTD for top voxels were more spread by about 7°C compared to all voxels) (Figure 5a,c). Otherwise, LAD and Gsmax did not influence the difference in mean LTD between the top and all voxels (Figure 5e,g). A similar trend was found for the variance in the LTDs: low wind speed and high irradiance generated large deviations between top and all voxels, and all voxels had a higher thermal variance than top voxels under these two specific circumstances (Figure 5b,d). Both LAD and Gsmax did not cause particular deviations between top and all voxels (Figure 5f,h).

### 3.4 Comparing the LTDs of the canopy surface and the whole canopy

**Figure 6:**
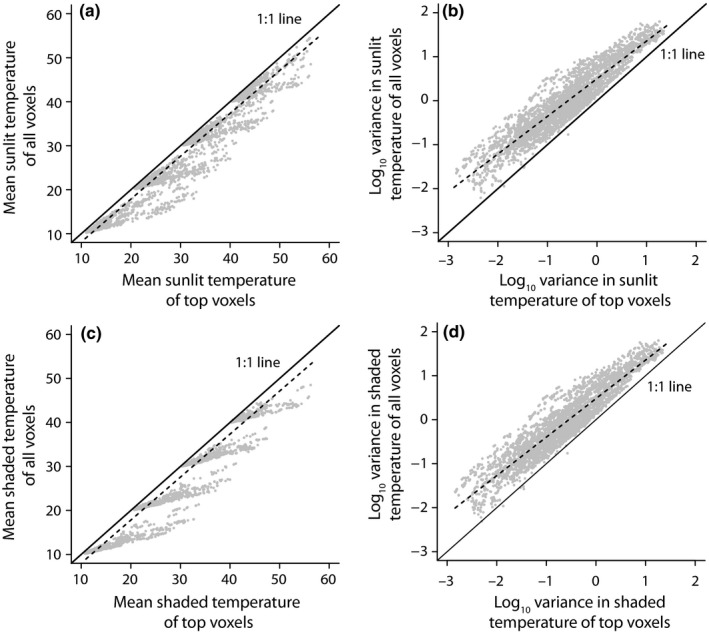
Regression analysis of mean temperature and variance of all voxels in the continuous canopy as function of top‐viewed voxels for both sunlit (a, b) and shaded (c, d) portions of voxels. Color‐coded versions of these figures are available in Appendices S4–S7, which show the effects of four other main variables (T_air_, wind, irradiance, and LAD) on these relationships (Figure 6a, linear regression: *p* < .001, *R* ^2^ = .95, *y* = 0.97*x* − 1.62; Figure 6b: *p* < .001, *R* ^2^ = .87, *y* = 0.87*x* + 0.50; Figure 6c: *p* < .001, *y* = 0.98*x* − 1.75; Figure 6d: *p* < .001, *R* ^2^ = .87, *y* = 0.87*x* + 0.48)

Using the mean and variance of top voxels as predictors, we were able to estimate the mean and variance of all voxels in the continuous canopy with fairly high confidence (Figure 6). Not surprisingly, top voxels overestimated mean leaf temperatures and underestimated thermal variance, throughout the entire canopy (Figure 6). However, in line with the results of the Morris analysis (see above), the correlation between top and all voxels was defined primarily by the most influential parameters: air temperature and wind speed for mean sunlit leaf temperature (Appendix S4) and LAD for the variance of sunlit leaf temperature (Appendix S6). For example, very low wind speeds generated a poor match between top voxels and all voxels, and high LAD values ameliorated the ability of top voxels to predict the thermal variance for all voxels.

## 4 DISCUSSION

Plant canopies are complex, heterogeneous spaces (Finnigan, 2000; Sinoquet et al., 2001; Urban et al., 2012). Leaf temperature distributions (LTDs) in plant canopies strongly influence local distributions and performance of small organisms, including phytopathogens, insect herbivores, and their predators and parasitoids (Bernard et al., 2013; Chelle, 2005; Pincebourde & Woods, 2012; Pincebourde et al., 2007), and LTDs can play a role in the responses of these organisms to environmental changes (Pincebourde, Murdock, Vickers, & Sears, 2016; Pincebourde & Suppo, 2016). Our sensitivity analysis of the RATP model indicates, not surprisingly, that climatic parameters have strong effects on mean leaf temperature at the canopy scale. This is because leaves are strongly coupled to atmospheric conditions, in particular to air temperature. However, we also found that structural parameters, especially leaf area density (LAD), can influence both the mean and spatial variance of temperatures, in some cases being more important than climatic parameters such as relative humidity. Finally, our results indicate that leaf physiological parameters are of minor importance compared to structural and climatic parameters in setting the leaf temperature distributions at the canopy scale. This result contrasts strongly with the relative attention paid in the literature to the consequences for leaf temperatures of, for example, leaf transpiration vs. leaf angle distribution. Overall, these interactions between parameters and their ranking in the Morris analysis make it possible to infer forest canopy LTDs from the LTDs measured or simulated at the surface of the canopy cover over a reasonable range of parameter values. This conclusion suggests that remote sensing data can be used to estimate 3D patterns of temperature variation from 2D images of vegetation surface temperatures.

Climate parameters also influenced variance in temperature. Intracanopy variance in temperature is directly generated by irradiance interacting with structural aspects of the canopy (but see below). At low irradiance, variance falls to low levels; at high irradiance, variance is magnified by irradiance attenuation through the canopy (Ngao et al., 2017). The ratio of diffuse to direct irradiance (parameter Kt) also affects the variance of canopy LTDs, but this effect is much less studied compared to others (Urban et al., 2012). More diffuse irradiance homogenizes leaf temperatures because shaded leaves receive relatively more radiative energy. Moreover, an increase in the diffuse portion is normally associated with cloud cover, which implies that the leaves exposed to the (overcast) sun are receiving less radiation at the same time (i.e., there is convergence of incoming irradiance for shaded and sunlit leaves) (Urban et al., 2012). Other climatic parameters may also influence the thermal variance. Wind speed generally is higher at the surface of canopies than inside, and this process is included in the RATP biophysical model (Sinoquet et al., 2001), although turbulent regimes in forest canopies can generate complex flow (Finnigan, 2000). The wind effect depends on the density of foliage, which explains why σ is high for this factor compared to others when looking at the thermal variance.

Importantly, structural parameters (especially leaf area density, LAD) were, in most cases, more important than physiological parameters. Their effects were strongest on the variance and autocorrelation indices of LTDs, meaning that architectural traits strongly influence the magnitude and configuration of spatial heterogeneity in leaf temperatures across canopy forest covers (see also Ref. (Scherrer et al., 2011)). Here too, this result emerges from interactions between irradiance and structure. Greater LAD means that much of the incoming irradiance is intercepted at the top of the tree, generating larger gradients and more aggregated temperatures. In addition, the position of the sun in the sky relative to the orientation and inclination of the leaf surfaces determines how much radiative energy is intercepted by these surfaces (geometric relationships—see Ref. (Oke, 2002)). Therefore, a high diversity of leaf angles should produce a high diversity of leaf temperatures spread throughout the canopy. Surprisingly, however, the effects of structural parameters exceeded the influence of physiological parameters. Although transpiration rate is well known to depress leaf temperatures (Campbell & Norman, 1998; Potter, Davidowitz, & Woods, 2009), the size of this effect was small compared to the effect of irradiation on the leaf heat budget at the canopy scale. This implies that changing stomatal parameters should induce changes locally in leaf surface temperatures, but that, taken at the global scale, the overall leaf surface distribution remains about the same. This is interpreted as the individual leaf temperature varying within the range corresponding to the current leaf temperature distribution. The interaction with other climatic factors such as wind further weakens the influence of transpiration; under high wind speed, changes in transpiration rate or any other physiological parameter do not induce large shifts in leaf temperature.

Our results are influenced by limitations inherent to the RATP model and, more generally, to the Morris analysis. First, the RATP model discretizes canopies into voxels (Sinoquet et al., 2001), an approach that necessarily homogenizes variables within voxels and thereby ignores variation at finer scales (Caillon et al., 2014; Saudreau et al., 2017). Discretization is necessary however to lower computational time and to allow the application of Beer's law to calculate the attenuation coefficient of irradiance across the forest canopy (Sinoquet et al., 2001). In addition, our aim was to analyze LTD at the scale of canopies, not at the scale of individual leaves (for leaf‐scale variation, see Saudreau et al., 2017), although the two scales are inter‐related and can be combined (Pincebourde & Suppo, 2016). Remote sensing and most plant–atmosphere exchange models are based on local/canopy scale processes, and our aim was to quantify the relative influence of the main climatic, structural, and physiological parameters in the same context as do these approaches. Second, the Morris approach is designed to estimate main effects of (μ*) and interactions among (σ) all parameters across the entire parameter space, and it does not account for potential correlations among variables that may occur in the real world (e.g., high humidity often co‐occurs with low irradiance). Thus, a more focused sensitivity analysis, in a subset of parameter space using predetermined correlation structures, may provide a different ranking of the more influential parameters. The advantage of the Morris analysis is that it provides a global view by examining the entire parameter space.

The main consequence of the interplay among canopy parameters, and of their relative influence on canopy LTDs, is that the temperatures measured at the surface of canopies are a reasonable proxy of the LTD within the entire continuous canopy. Our results indicate that over a large range of parameter space, the LTDs of top‐viewed voxels approximate the LTDs of all voxels. Nevertheless, low wind speeds and high irradiance both result in strong deviations between the LTDs of top‐viewed voxels and all voxels, because under these conditions, the temperature of leaves at the top of the tree canopies increases markedly relative to other leaves. Still, we estimate that the prediction error of the median leaf temperature is <3°C under these extreme conditions (but see Figure 6). These deviations are reflected both in mean leaf temperature and, to a lesser extent, the thermal variance. Overall, the least influential parameters from the Morris analysis (e.g., stomatal conductance) did not induce deviation between top voxels and all voxels.

Our results support several recommendations for biologists interested in thermal ecology and the global change biology of forest insects. First, we need more data on plant architectures (Barthélémy & Caraglio, 2007) across latitudes, altitudes, and biomes. Currently, detailed architectural traits are available primarily from crops and orchards (Godin & Sinoquet, 2005; Sinoquet et al., 2009) but not from single species or mixed‐species stands in the wild (see Ref. (Farque, Sinoquet, & Colin, 2001) for an exception). Currently, therefore, it is not possible to compare our results on apple with similar approaches on forest trees. The well‐known TRY database (Kattge et al., 2011), which lists numerous plant traits for a large number of species across the world, contains architectural traits, but they are underrepresented compared to physiological parameters. Several tools exist to measure basic architectural traits, including electromagnetic digitizers (Godin, Costes, & Sinoquet, 1999; Sinoquet, Thanisawanyangkura, Mabrouk, & Kasemsap, 1998), LIDAR scanning (Béland, Widlowski, & Fournier, 2014), and orthophotography for simple canopies (Rich, 1990). We therefore call for additional use of these tools to collect architectural traits on diverse groups of plant species. Second, the overall range of surface temperatures (3D) available to small organisms living on leaf surfaces can be approximated from infrared thermography of canopy surfaces (2D). Remote sensing satellites, unmanned aerial vehicles (UAVs), or crane towers equipped with infrared sensors are now providing such 2D temperature data for a wide range of plant species across latitudes (Dong, Prentice, Harrison, Song, & Zhang, 2017; Faye et al., 2017; Leuzinger & Körner, 2007). Our results suggest that remote sensing data at adequate spatial resolutions can be used to predict the distribution and performance of plant‐associated organisms within forest canopies.

## CONFLICT OF INTEREST

None declared.

## AUTHOR CONTRIBUTIONS

HAW, MS, and SP conceived of the project; MS parameterized and ran the RATP model and Morris analyses; HAW did additional analysis; HAW and MS constructed the figures; and HAW, MS, and SP wrote the manuscript.

## DATA ACCESSIBILITY

Data from the article will be made available in an online repository once the article is accepted.

## References

[ece34046-bib-0001] Bailey, B. N. , Stoll, R. , Pardyjak, E. R. , & Miller, N. E. (2016). A new three‐dimensional energy balance model for complex plant canopy geometries: Model development and improved validation strategies. Agricultural and Forest Meteorology, 218–219, 146–160. https://doi.org/10.1016/j.agrformet.2015.11.021

[ece34046-bib-0002] Baldocchi, D. D. , Wilson, K. B. , & Gu, L. (2002). How the environment, canopy structure and canopy physiological functioning influence carbon, water and energy fluxes of a temperate broad‐leaved deciduous forest–an assessment with the biophysical model CANOAK. Tree Physiology, 22, 1065–1077. https://doi.org/10.1093/treephys/22.15-16.1065 12414367 10.1093/treephys/22.15-16.1065

[ece34046-bib-0003] Bale, J. S. , Masters, G. J. , Hodkinson, I. D. , Awmack, C. , Bezemer, T. M. , Brown, V. K. , … Whittaker, J. B. (2002). Herbivory in global climate change research: Direct effects of rising temperature on insect herbivores. Global Change Biology, 8, 1–16. https://doi.org/10.1046/j.1365-2486.2002.00451.x

[ece34046-bib-0004] Barthélémy, D. , & Caraglio, Y. (2007). Plant architecture: A dynamic, multilevel and comprehensive approach to plant form, structure and ontogeny. Annals of Botany, 99, 375–407. https://doi.org/10.1093/aob/mcl260 17218346 10.1093/aob/mcl260 PMC2802949

[ece34046-bib-0005] Bauerle, W. L. , Bowden, J. D. , Wang, G. G. , & Shahba, M. A. (2009). Exploring the importance of within‐canopy spatial temperature variation on transpiration predictions. Journal of Experimental Botany, 60, 3665–3676. https://doi.org/10.1093/jxb/erp206 19561047 10.1093/jxb/erp206 PMC2736884

[ece34046-bib-0006] Béland, M. , Widlowski, J. L. , & Fournier, R. A. (2014). A model for deriving voxel‐level tree leaf area density estimates from ground‐based LiDAR. Environmental Modelling and Software, 51, 184–189. https://doi.org/10.1016/j.envsoft.2013.09.034

[ece34046-bib-0007] Benedetti‐Cecchi, L. , Bertocci, I. , Vaselli, S. , & Maggi, E. (2006). Temporal variance reverses the impact of high mean intensity of stress in climate change experiments. Ecology, 87, 2489–2499. https://doi.org/10.1890/0012-9658(2006)87[2489:TVRTIO]2.0.CO;2 17089658 10.1890/0012-9658(2006)87[2489:tvrtio]2.0.co;2

[ece34046-bib-0008] Bernard, F. , Sache, I. , Suffert, F. , & Chelle, M. (2013). The development of a foliar fungal pathogen does react to leaf temperature!. New Phytologist, 198, 232–240. https://doi.org/10.1111/nph.12134 23373986 10.1111/nph.12134

[ece34046-bib-0009] Bonan, G. B. (1996). A Land Surface Model (LSM Version 1.0) For Ecological Hydrological and Atmospheric Studies: Technical Description and User's Guide. *NCAR Technical Note*, 156.

[ece34046-bib-0010] Brent, R. P. (1974). Algorithms for minimization without derivatives. IEEE Transactions on Automatic Control, 19, 632–633.

[ece34046-bib-0011] Caillon, R. , Suppo, C. , Casas, J. , Woods, H. A. , & Pincebourde, S. (2014). Warming decreases thermal heterogeneity of leaf surfaces: Implications for behavioural thermoregulation by arthropods. Functional Ecology, 28, 1449–1458. https://doi.org/10.1111/1365-2435.12288

[ece34046-bib-0012] Caldwell, M. M. , Meister, H. P. , Tenhunen, J. D. , & Lange, O. L. (1986). Canopy structure, light microclimate and leaf gas exchange of *Quercus coccifera* L. in a Portuguese macchia: Measurements in different canopy layers and simulations with a canopy model. Trees, 1, 25–41.

[ece34046-bib-0013] Campbell, G. S. , & Norman, J. M. (1998). An introduction to environmental biophysics. New York: Springer https://doi.org/10.1007/978-1-4612-1626-1

[ece34046-bib-0014] Campolongo, F. , Cariboni, J. , & Saltelli, A. (2007). An effective screening design for sensitivity analysis of large models. Environmental Modelling and Software, 22, 1509–1518. https://doi.org/10.1016/j.envsoft.2006.10.004

[ece34046-bib-0015] Chelle, M. (2005). Phylloclimate or the climate perceived by individual plant organs: What is it? How to model it? What for? The New Phytologist, 166, 781–790. https://doi.org/10.1111/j.1469-8137.2005.01350.x 15869641 10.1111/j.1469-8137.2005.01350.x

[ece34046-bib-0016] Collatz, G. J. , Ball, J. T. , Grivet, C. , & Berry, J. A. (1991). Physiological and environmental regulation of stomatal conductance, photosynthesis and transpiration: A model that includes a laminar boundary layer. Agricultural and Forest Meteorology, 54, 107–136. https://doi.org/10.1016/0168-1923(91)90002-8

[ece34046-bib-0017] Dai, Y. , Dickinson, R. E. , & Wang, Y. P. (2004). A two‐big‐leaf model for canopy temperature, photosynthesis, and stomatal conductance. Journal of Climate, 17, 2281–2299. https://doi.org/10.1175/1520-0442(2004)017<2281:ATMFCT>2.0.CO;2

[ece34046-bib-0018] Daudet, F. A. , Le Roux, X. , Sinoquet, H. , & Adam, B. (1999). Wind speed and leaf boundary layer conductance variation within tree crown consequences on leaf‐to‐atmosphere coupling and tree functions. Agricultural and Forest Meteorology, 97, 171–185. https://doi.org/10.1016/S0168-1923(99)00079-9

[ece34046-bib-0019] Dillon, M. E. , Woods, H. A. , Wang, G. , Fey, S. B. , Vasseur, D. A. , Telemeco, R. S. , … Pincebourde, S. (2016). Life in the frequency domain: The biological impacts of changes in climate variability at multiple time scales. Integrative and Comparative Biology, 56, 14–30. https://doi.org/10.1093/icb/icw024 27252201 10.1093/icb/icw024

[ece34046-bib-0020] Dong, N. , Prentice, I. C. , Harrison, S. P. , Song, Q. H. , & Zhang, Y. P. (2017). Biophysical homoeostasis of leaf temperature: A neglected process for vegetation and land‐surface modelling. Global Ecology and Biogeography, 26, 998–1007. https://doi.org/10.1111/geb.12614

[ece34046-bib-0021] Farque, L. , Sinoquet, H. , & Colin, F. (2001). Canopy structure and light interception in *Quercus petraea* seedlings in relation to light regime and plant density. Tree physiology, 21, 1257–1267. https://doi.org/10.1093/treephys/21.17.1257 11696413 10.1093/treephys/21.17.1257

[ece34046-bib-0022] Faye, E. , Rebaudo, F. , Carpio, C. , Herrera, M. , & Dangles, O. (2017). Does heterogeneity in crop canopy microclimates matter for pests? Evidence from aerial high‐resolution thermography. Agriculture, Ecosystems and Environment, 246, 124–133. https://doi.org/10.1016/j.agee.2017.05.027

[ece34046-bib-0023] Faye, E. , Rebaudo, F. , Yánez‐Cajo, D. , Cauvy‐Fraunié, S. , & Dangles, O. (2016). A toolbox for studying thermal heterogeneity across spatial scales: From unmanned aerial vehicle imagery to landscape metrics. Methods in Ecology and Evolution, 7, 437–446. https://doi.org/10.1111/2041-210X.12488

[ece34046-bib-0024] Finnigan, J. (2000). Turbulence in plant canopies. Annual Review of Fluid Mechanics, 32, 519–571. https://doi.org/10.1146/annurev.fluid.32.1.519

[ece34046-bib-0025] Flerchinger, G. N. , Reba, M. L. , Link, T. E. , & Marks, D. (2016). Modeling temperature and humidity profiles within forest canopies. Agricultural and Forest Meteorology, 213, 251–262.

[ece34046-bib-0026] Fortin, M.‐J. , Drapeau, P. , & Legendre, P. (1989). Spatial autocorrelation and sampling design in plant ecology. Vegetatio, 83, 209–222. https://doi.org/10.1007/BF00031693

[ece34046-bib-0027] Godin, C. , Costes, E. , & Sinoquet, H. (1999). A method for describing plant architecture which integrates topology and geometry. Annals of Botany, 84, 343–357. https://doi.org/10.1006/anbo.1999.0923

[ece34046-bib-0028] Godin, C. , & Sinoquet, H. (2005). Functional‐structural plant modelling. New Phytologist, 166, 705–708. https://doi.org/10.1111/j.1469-8137.2005.01445.x 15869632 10.1111/j.1469-8137.2005.01445.x

[ece34046-bib-0029] Helliker, B. R. , & Richter, S. L. (2008). Subtropical to boreal convergence of tree‐leaf temperatures. Nature, 454, 511–514. https://doi.org/10.1038/nature07031 18548005 10.1038/nature07031

[ece34046-bib-0030] Iziomon, M. G. , Mayer, H. , & Matzarakis, A. (2003). Downward atmospheric longwave irradiance under clear and cloudy skies: Measurement and parameterization. Journal of Atmospheric and Solar‐Terrestrial Physics, 65, 1107–1116. https://doi.org/10.1016/j.jastp.2003.07.007

[ece34046-bib-0200] Jarvis, P. G. (1976). The interpretation of the variations in leaf water potential and stomatal conductance found in canopies in the field. Phil. Trans. R. Soc. Lond. B, 273(927), 593–610. 10.1098/rstb.2014.0311 PMC4360119 25750234

[ece34046-bib-0031] Jones, H. G. (1992) Plants and microclimate: A quantitative approach to environmental plant physiolog. Cambridge: Cambridge University Press.

[ece34046-bib-0032] Jumars, P. A. , Thistle, D. , & Jones, M. L. (1977). Detecting 2‐dimensional spatial structure in biological data. Oecologia, 28, 109–123. https://doi.org/10.1007/BF00345246 28309009 10.1007/BF00345246

[ece34046-bib-0033] Kattge, J. , Díaz, S. , Lavorel, S. , Prentice, I. C. , Leadley, P. , Bönisch, G. , … Wirth, C. (2011). TRY ‐ a global database of plant traits. Global Change Biology, 17, 2905–2935. https://doi.org/10.1111/j.1365-2486.2011.02451.x

[ece34046-bib-0034] Kearney, M. , Shine, R. , & Porter, W. P. (2009). The potential for behavioral thermoregulation to buffer ‘cold‐blooded’ animals against climate warming. Proceedings of the National Academy of Sciences of the United States of America, 106, 3835–3840. https://doi.org/10.1073/pnas.0808913106 19234117 10.1073/pnas.0808913106 PMC2656166

[ece34046-bib-0035] King, D. M. , & Perera, B. J. C. (2013). Morris method of sensitivity analysis applied to assess the importance of input variables on urban water supply yield ‐ A case study. Journal of Hydrology, 477, 17–32. https://doi.org/10.1016/j.jhydrol.2012.10.017

[ece34046-bib-0036] Kingsolver, J. G. (2009). The well‐temperatured biologist. American Naturalist, 174, 755–768. 10.1086/648310 19857158

[ece34046-bib-0037] Kobza, J. , & Edwards, G. E. (1987). Influences of leaf temperature on photosynthetic carbon metabolism in wheat. Plant Physiology, 83, 69–74. https://doi.org/10.1104/pp.83.1.69 16665218 10.1104/pp.83.1.69 PMC1056301

[ece34046-bib-0038] Leuning, R. , Kelliher, F. M. , de Pury, D. G. G. , & Schulze, E. D. (1995). Leaf nitrogen, photosynthesis, conductance and transpiration: Scaling from leaves to canopies. Plant, Cell and Environment, 18, 1183–1200. https://doi.org/10.1111/j.1365-3040.1995.tb00628.x

[ece34046-bib-0039] Leuzinger, S. , & Körner, C. (2007). Tree species diversity affects canopy leaf temperatures in a mature temperate forest. Agricultural and Forest Meteorology, 146, 29–37. https://doi.org/10.1016/j.agrformet.2007.05.007

[ece34046-bib-0040] Linacre, E. T. (1967). Further notes on a feature of leaf and air temperatures. Theoretical and Applied Climatology, 15, 422–436.

[ece34046-bib-0041] Lintunen, A. , Sievänen, R. , Kaitaniemi, P. , & Perttunen, J. (2011). Models of 3D crown structure for Scots pine (*Pinus sylvestris*) and silver birch (*Betula pendula*) grown in mixed forest. Forest Research, 1794, 1779–1794.

[ece34046-bib-0042] Michaletz, S. T. , Weiser, M. D. , McDowell, N. G. , Zhou, J. , Kaspari, M. , Helliker, B. R. , & Enquist, B. J. (2016). The energetic and carbon economic origins of leaf thermoregulation. Nature Plants, 2, 16129 https://doi.org/10.1038/nplants.2016.129 27548589 10.1038/nplants.2016.129

[ece34046-bib-0043] Monteith, J. , & Unsworth, M. (2008). Principles of environmental physics. London, UK: Academic Press.

[ece34046-bib-0044] Morris, M. D. (1991). Factorial sampling plans for preliminary computational experiments. Technometrics, 33, 161 https://doi.org/10.1080/00401706.1991.10484804

[ece34046-bib-0045] Ngao, J. , Adam, B. , & Saudreau, M. (2017). Intra‐crown spatial variability of leaf temperature and stomatal conductance enhanced by drought in apple tree as assessed by the RATP model. Agricultural and Forest Meteorology, 237–238, 340–354. https://doi.org/10.1016/j.agrformet.2017.02.036

[ece34046-bib-0046] Niinemets, Ü. , Keenan, T. F. , & Hallik, L. (2015). A worldwide analysis of within‐canopy variations in leaf structural, chemical and physiological traits across plant functional types. New Phytologist, 205, 973–993. https://doi.org/10.1111/nph.13096 25318596 10.1111/nph.13096 PMC5818144

[ece34046-bib-0047] Nobel, P. S. (1999). Physicochemical & environmental plant physiology. New York, NY: Academic Press.

[ece34046-bib-0048] Oke, T. R. (2002). Boundary Layer Climates. London, UK: Routledge.

[ece34046-bib-0049] Pincebourde, S. , Murdock, C. C. , Vickers, M. , & Sears, M. W. (2016). Fine‐scale microclimatic variation can shape the responses of organisms to global change in both natural and urban environments. Integrative and Comparative Biology, 56, 45–61. https://doi.org/10.1093/icb/icw016 27107292 10.1093/icb/icw016

[ece34046-bib-0050] Pincebourde, S. , Sinoquet, H. , Combes, D. , & Casas, J. (2007). Regional climate modulates the canopy mosaic of favourable and risky microclimates for insects. Journal of Animal Ecology, 76, 424–438. https://doi.org/10.1111/j.1365-2656.2007.01231.x 17439460 10.1111/j.1365-2656.2007.01231.x

[ece34046-bib-0051] Pincebourde, S. , & Suppo, C. (2016). The vulnerability of tropical ectotherms to warming is modulated by the microclimatic heterogeneity. Integrative and Comparative Biology, 56, 85–97. https://doi.org/10.1093/icb/icw014 27371561 10.1093/icb/icw014

[ece34046-bib-0052] Pincebourde, S. , & Woods, H. A. (2012). Climate uncertainty on leaf surfaces: The biophysics of leaf microclimates and their consequences for leaf‐dwelling organisms. Functional Ecology, 26, 844–853. https://doi.org/10.1111/j.1365-2435.2012.02013.x

[ece34046-bib-0053] Potter, K. A. , Davidowitz, G. , & Woods, H. A. (2009). Insect eggs protected from high temperatures by limited homeothermy of plant leaves. Journal of Experimental Biology, 212, 3448–3454. https://doi.org/10.1242/jeb.033365 19837886 10.1242/jeb.033365

[ece34046-bib-0054] Pyles, R. D. , Weare, B. C. , & Pawu, K. T. (2000). The UCD advanced canopy‐atmosphere‐soil algorithm: Comparisons with observations from different climate and vegetation regimes. Quarterly Journal of the Royal Meteorological Society, 126, 2951–2980. https://doi.org/10.1002/(ISSN)1477-870X

[ece34046-bib-0055] Reindl, D. T. , Beckman, W. A. , & Duffie, J. A. (1990). Diffuse fraction correlations. Solar Energy, 45, 1–7. https://doi.org/10.1016/0038-092X(90)90060-P

[ece34046-bib-0056] Rich, P. M. (1990). Characterizing plant canopies with hemispherical photographs. Remote Sensing Reviews, 5, 13–29. https://doi.org/10.1080/02757259009532119

[ece34046-bib-0057] Saudreau, M. , Ezanic, A. , Adam, B. , Caillon, R. , Walser, P. , & Pincebourde, S. (2017). Temperature heterogeneity over leaf surfaces: The contribution of the lamina microtopography. Plant, Cell and Environment, 40, 2174–2188. 10.1111/pce.13026 28710812

[ece34046-bib-0058] Saudreau, M. , Pincebourde, S. , Dassot, M. , Adam, B. , Loxdale, H. D. , & Biron, D. G. (2013). On the canopy structure manipulation to buffer climate change effects on insect herbivore development. Trees, 27, 239–248. https://doi.org/10.1007/s00468-012-0791-7

[ece34046-bib-0059] Scherrer, D. , Bader, M. K.‐F. , & Körner, C. (2011). Drought‐sensitivity ranking of deciduous tree species based on thermal imaging of forest canopies. Agricultural and Forest Meteorology, 151, 1632–1640. https://doi.org/10.1016/j.agrformet.2011.06.019

[ece34046-bib-0060] Sears, M. W. , Angilletta, M. J. , Schuler, M. S. , Borchert, J. , Dilliplane, K. F. , Stegman, M. , … Mitchell, W. A. (2016). Configuration of the thermal landscape determines thermoregulatory performance of ectotherms. Proceedings of the National Academy of Sciences of the United States of America, 113, 10595–10600. https://doi.org/10.1073/pnas.1604824113 27601639 10.1073/pnas.1604824113 PMC5035910

[ece34046-bib-0061] Sellers, P. J. (1997). Modeling the exchanges of energy, water, and carbon between continents and the atmosphere. Science, 275, 502–509. https://doi.org/10.1126/science.275.5299.502 8999789 10.1126/science.275.5299.502

[ece34046-bib-0062] Sinoquet, H. , Le Roux, X. , Adam, B. , Ameglio, T. , & Daudet, F. A. (2001). RATP: A model for simulating the spatial distribution of radiation absorption, transpiration and photosynthesis within canopies: Application to an isolated tree crown. Plant, Cell and Environment, 24, 395–406.

[ece34046-bib-0063] Sinoquet, H. , Pincebourde, S. , Adam, B. , Donès, N. , Phattaralerphong, J. , Combes, D. , … Casas, J. (2009). 3‐D maps of tree canopy geometries at leaf scale. Ecology, 90, 283–283. https://doi.org/10.1890/08-0179.1

[ece34046-bib-0064] Sinoquet, H. , Sonohat, G. , Phattaralerphong, J. , & Godin, C. (2005). Foliage randomness and light interception in 3‐D digitized trees: An analysis from multiscale discretization of the canopy. Plant, Cell and Environment, 28, 1158–1170. https://doi.org/10.1111/j.1365-3040.2005.01353.x

[ece34046-bib-0065] Sinoquet, H. , Thanisawanyangkura, S. , Mabrouk, H. , & Kasemsap, P. (1998). Characterization of the light environment in canopies using 3D digitising and image processing. Annals of Botany, 82, 203–212. https://doi.org/10.1006/anbo.1998.0665

[ece34046-bib-0066] Sunday, J. M. , Bates, A. E. , Kearney, M. R. , Colwell, R. K. , Dulvy, N. K. , Longino, J. T. , & Huey, R. B. (2014). Thermal‐safety margins and the necessity of thermoregulatory behavior across latitude and elevation. Proceedings of the National Academy of Sciences of the United States of America, 111, 5610–5615. https://doi.org/10.1073/pnas.1316145111 24616528 10.1073/pnas.1316145111 PMC3992687

[ece34046-bib-0067] Tuzet, A. , Perrier, A. , & Leuning, R. (2003). A coupled model of stomatal conductance, photosynthesis. Plant, Cell and Environment, 26, 1097–1116. https://doi.org/10.1046/j.1365-3040.2003.01035.x

[ece34046-bib-0068] Urban, O. , Klem, K. , Ač, A. , Havránková, K. , Holišová, P. , Navrátil, M. , … Grace, J. (2012). Impact of clear and cloudy sky conditions on the vertical distribution of photosynthetic CO 2 uptake within a spruce canopy. Functional Ecology, 26, 46–55. https://doi.org/10.1111/j.1365-2435.2011.01934.x

[ece34046-bib-0069] Vasseur, D. A. , DeLong, J. P. , Gilbert, B. , Greig, H. S. , Harley, C. D. G. , McCann, K. S. , … O’ Connor, M. I. (2014). Increased temperature variation poses a greater risk to species than climate warming. Proceedings of the Royal Society B, 281, 20132612. 24478296 10.1098/rspb.2013.2612 PMC3924069

[ece34046-bib-0070] Wang, Y. P. , & Jarvis, P. G. (1990). Description and validation of an array model ‐ MAESTRO. Agricultural and Forest Meteorology, 51, 257–280. https://doi.org/10.1016/0168-1923(90)90112-J

[ece34046-bib-0071] Wang, W. M. , Li, Z. L. , & Su, H. B. (2007). Comparison of leaf angle distribution functions: Effects on extinction coefficient and fraction of sunlit foliage. Agricultural and Forest Meteorology, 143, 106–122. https://doi.org/10.1016/j.agrformet.2006.12.003

[ece34046-bib-0072] Wohlfahrt, G. (2004). Modelling fluxes and concentrations of CO2, H2O and sensible heat within and above a mountain meadow canopy: A comparison of three Lagrangian models and three parameterisation options for the Lagrangian time scale. Boundary‐Layer Meteorology, 113, 43–80. https://doi.org/10.1023/B:BOUN.0000037326.40490.1f

[ece34046-bib-0073] Woods, H. A. , Dillon, M. E. , & Pincebourde, S. (2015). The roles of microclimatic diversity and of behavior in mediating the responses of ectotherms to climate change. Journal of Thermal Biology, 54, 86–97. 26615730 10.1016/j.jtherbio.2014.10.002

