# Supplementary material for: Structure is more important than physiology for estimating intracanopy distributions of leaf temperatures
Source: Ecol Evol. 2018 Apr 27;8(10):5206–18. doi: 10.1002/ece3.4046 (PMC5980536; doi:10.1002/ece3.4046)
Supplement: Supplementary file 1 [file ECE3-8-5206-s001.docx]

**Supporting Information**

***S1. The RAPT model: modelling radiation interception by canopies.***

The RATP model (Radiation Absorption, Transpiration and Photosynthesis) was designed to describe the spatial distribution of radiation, transpiration and photosynthesis within plant canopies. The full model is described in (Sinoquet *et al.* 2001) and other publications since then (Pincebourde *et al.* 2007; Massonnet *et al.* 2008; Saudreau *et al.* 2013). Here, the sub-model simulating the interception of solar radiation by leaves at the intra-canopy scale was used. The model is based on the Beer’s law, the canopy being treated as a turbid medium. Inputs for this model are the canopy geometry, optical properties of leaves and soil surface and the climatic driving variables. The canopy geometry is described by discretizing the space into a grid of 3D cubic cells (voxels). Each cell might be empty or characterized by the area density of a given plant component, according to 3D digitizing data. Here, one canopy component was defined, the leaves.

The radiative transfer sub-model is aimed at (i) sending beams into the canopy according to the directional distribution of incident radiation, by taking into account for the sun direction and the distribution of incident radiation into direct and diffuse radiation; radiance distribution for diffuse radiation is assumed to obey the standard overcast sky (Moon & Spencer 1942); (ii) identifying the 3D sequence of cells crossed by any light beam; (iii) determining the beam path length within each crossed cell; and (iv) applying Beer’s law to calculate beam extinction within each crossed voxel (Sinoquet *et al.* 2001). Radiation sources are the sky, including direct and diffuse (i.e. scattered by clouds and atmospheric gases) fraction of incident radiation, as well as foliage components and soil surface which scatter a fraction of radiation they intercept. The flux of radiation (W/m^2^) intercepted by a leaf, in cell *k*, in sunlit and shaded area is respectively

$$I_{k}^{sun}=I_{k}^{dc}+I_{k}^{df}+I_{k}^{L-sc}+I_{k}^{S-sc}$$

$$I_{k}^{shade}=I_{k}^{df}+I_{k}^{L-sc}+I_{k}^{S-sc}$$

where $I_{k}^{dc}$ is the flux of direct radiation intercepted by a leaf in cell *k*, $I_{k}^{df}$ is the intercepted flux of diffuse radiation, $I_{k}^{L-sc}$ is the intercepted flux of radiation scattered by neighboring leaves, and $I_{k}^{S-sc}$ is the intercepted flux of radiation scattered by soil surface (all in W/m^2^). The terms in equations (1) and (2) are detailed below.

The direct beam light is intercepted only by the sunlit leaf area. The flux of direct solar radiation intercepted by a leaf in cell *k* depends on the direction of the beam light ($\Omega$) and is calculated from

$$I_{k}^{dc}=I_{bo}\frac{G_{k}\left(\Omega\right)}{\sin\left(h_{sun}\right)}$$

where $I_{bo}$ is the flux of direct radiation on a horizontal plane, $h_{sun}$ is sun elevation, and $G_{k}\left(\Omega\right)$ is the projection of leaf area in cell *k* onto a plane perpendicular to beam light direction ($\Omega$) and depends on inclination angle distribution of leaves within the cell *k*.

The flux of diffuse radiation intercepted by a leaf in cell *k* is

$$I_{k}^{df}=I_{do} C_{do\to k}$$

where $I_{do}$ is the flux of incident diffuse radiation on a horizontal plane. $C_{do\to k}$ is the radiation exchange coefficient between the sky, which emits diffuse radiation, and leaves in cell *k*. The computation used to derived radiation exchange coefficients is given in Sinoquet *et al.* (2001).

The flux of radiation scattered by leaves in each grid cell and intercepted by a leaf in cell *k* is computed from

$$I_{k}^{L-sc}=\sum_{k'=1}^{K} I_{k'} \rho_{L} C_{k'\to k}$$

where $I_{k'}$ is the flux of radiation intercepted by leaves in cell *k’* (there are *K* cells), $\rho_{L}$ is the scattering coefficient of leaves (obtained by summing transmittance and reflectance of leaves), and $C_{k'\to k}$ is the radiation exchange coefficient between leaves in cell *k’* and those in cell *k*.

The flux of radiations scattered by a soil unit surface *s* (there are *S* soil unit surfaces) and intercepted by leaves in cell *k* is determined by

$$I_{k}^{S-sc}=\sum_{s=1}^{S} I_{s} \rho_{s} C_{s\to k}$$

where $I_{s}$ is the flux of radiation intercepted by the soil, $\rho_{s}$ is the scattering coefficient of the soil, and $C_{s\to k}$ is the coefficient of radiation exchange from soil to leaves in cell *k*.

This model for interception of solar radiation was computed for both photosynthetically active radiation (PAR, 400 to 700 nm waveband) and near infrared radiation (NIR, 700 to 2500 nm waveband). Outputs are fluxes of PAR and NIR intercepted by both sunlit and shaded areas in each 3D cell occupied by leaves.

### **Modelling the energy budget of a leaf**

We used published biophysical models to compute the energy balance of leaves (Campbell & Norman 1998; Nobel 1999; Pincebourde & Casas 2006). The energy budget model of component *j* (a leaf) in cell *k* is

$A_{jk}$ is the amount of surface of component *j* in cell *k*. $Rn_{j}$ is the net radiation budget of the component *j*, $E_{j}$ is the latent heat budget (i.e. heat lost during evaporation), and $H_{j}$ is the sensible heat budget (i.e. heat lost during conduction and convection mechanisms) (all in W/m^2^). Values of each term between brackets are positive or negative depending on the direction of the heat flux - gain or lost by the component, respectively. Energy storage by leaves was neglected (Nobel 1999; Pincebourde & Casas 2006). The terms of the energy budget are detailed below and are given for sunlit area only, as the equations also apply to compute energy budget in shaded area using parameters for shaded components.

The net radiation balance of component *j* in cell *k* is the sum of the radiative heat fluxes occurring in three wavebands, i.e. PAR, NIR and thermal infrared radiations (TIR), that is

$$Rn_{jk}^{sun}=a_{j}^{PAR}I_{k}^{sun,PAR}+a_{j}^{NIR}I_{k}^{sun,NIR}+a_{j}^{TIR}I_{k}^{sun,TIR}-2\varepsilon_{j} \sigma T_{jk}^{sun^{4}}$$

$a_{j}^{PAR}$, $a_{j}^{NIR}$, and $a_{j}^{TIR}$ are absorbance of component *j* in each waveband. According to Kirchhoff’s law, leaf absorbance in the TIR waveband equals emissivity, i.e. $a_{j}^{TIR}=\varepsilon_{j}=0.97$ (Campbell & Norman 1998). Fluxes $I_{k}^{sun,PAR}$ and $I_{k}^{sun,NIR}$ are computed from equation (1). The flux $I_{k}^{sun,TIR}$ is calculated from equation (1) assuming scattering coefficients of soil and component surfaces in TIR waveband are zero (Campbell & Norman 1998), and including the emitted TIR by neighboring leaves (in sunlit and shaded areas as well) and soil unit surfaces (see Sinoquet *et al.* 2001). The last term on the right of equation (8) corresponds to TIR emitted by component *j* in cell *k*. The amount of emitted TIR depends on the Stephan-Boltzman constant ($\sigma$= 5.67 10^-8^ W m^-2^ K^-4^) and on temperature$T_{j}$ of the component.

Each component *j* (leaves) loses water vapor through lower and upper surfaces. The latent heat lost during evaporation is

$$E_{jk}=\lambda g_{jv}^{sun}\left(\frac{e_{s}\left(T_{jk}^{sun}\right)-e_{a}}{p_{a}}\right)$$

where $\lambda$ is the latent heat of vaporization for water ($\lambda$ = 44 kJ/mol at 25°C), $g_{jv}^{sun}$ is the conductance for water vapor transfer of component *j* (mol m^-2^ s^-1^), $e_{s}\left(T_{jk}^{sun}\right)$ is the saturated water vapor pressure (Pa) at temperature $T_{jk}^{sun}$ (°C) of the component *j* in cell *k*, $e_{a}$ is the water vapor pressure in the air (Pa) and $p_{a}$ is the atmospheric pressure (101.3 x 10^3^ Pa). Water vapor pressure in the air was assumed to be constant within the tree canopy, as confirmed by (Daudet *et al.* 1999). In hypostomatous leaves, the conductance for water vapor transfer is calculated by combining conductance of the boundary layer ($g_{jva}^{sun}$) and conductance through upper (transpiration through epidermis) and lower (through stomata) sides of component *j* ($g_{jk}^{u,sun}$ and $g_{jk}^{st,sun}$ respectively), that is

$$g_{jv}^{sun}=\frac{0.5}{\frac{1}{g_{jva}^{sun}}+\frac{1}{g_{jk}^{u,sun}}}+\frac{0.5}{\frac{1}{g_{jva}^{sun}}+\frac{1}{g_{jk}^{st,sun}}}$$

We used the model of Jarvis (Jarvis 1976) according to which the effect of each climatic variable on stomatal conductance is independent from each over (non-synergetic interactions). Stomatal conductance was therefore calculated by

$$g_{jk}^{st,sun}=g_{j}^{\max} f_{j}^{1}\left(I_{jk}^{sun,PAR}\right) f_{j}^{2}\left(e_{s}\left(T_{jk}^{sun}\right)-e_{a}\right) f_{j}^{3}\left(T_{jk}^{sun}\right)$$

where $g_{j}^{\max}$is the maximal stomatal conductance (mol m^-2^ s^-1^), attained under specific levels of irradiance ($I_{jk}^{sun,PAR}$: µmol PAR m^-2^ s^-1^), water vapor pressure deficit of component *j* ($\left(e_{s}\left(T_{jk}^{sun}\right)-e_{a}\right)$: Pa) and component’s temperature ($T_{jk}^{sun}$: °C), and $f_{j}^{1}$, $f_{j}^{2}$, and $f_{j}^{3}$ are the functions describing the variations of the stomatal conductance relative to the maximal value following a change in irradiance level, water vapor pressure deficit and component temperature, respectively.

The sensible heat budget of sunlit leaves (subscript *L*) in cell *k* is given by

$$H_{Lk}=c_{p} g_{L}^{ha} \left(T_{Lk}^{sun}-T_{air}\right)$$

where $c_{p}$ is the specific heat of the air (29.3 J mol^-1^ °C^-1^), and $T_{air}$ is air temperature (K). Air temperature was assumed to be constant within the tree canopy. The leaf boundary layer conductance for heat under forced convection mechanisms ($g_{L}^{ha}$: mol m^-2^ s^-1^) was computed from local wind speed (i.e. wind speed at a leaf surface) using the method given in Pincebourde & Casas (Pincebourde & Casas 2006). Local wind speed was obtained from wind speed values taken above the canopy using the empirical relationships between wind speed attenuation and the total amount of foliage along wind vector given in Daudet *et al.* (Daudet *et al.* 1999).

The energy balance (equation 7) was solved for temperature using Brent’s method (Brent 1974). Outputs of the energy budget model are temperatures of leaves in sunlit and shaded areas within each 3D cell of the canopy ($T_{Lk}^{sun}$, $T_{Mk}^{sun}$, $T_{Lk}^{shade}$ and $T_{Mk}^{shade}$).

**References**

Brent, R.P. (1974) Algorithms for Minimization Without Derivatives. *IEEE Transactions on Automatic Control*, **19**, 632–633.

Campbell, G.S. & Norman, J.M. (1998) *An Introduction to Environmental Biophysics*. Springer.

Daudet, F.A., Le Roux, X., Sinoquet, H. & Adam, B. (1999) Wind speed and leaf boundary layer conductance variation within tree crown consequences on leaf-to-atmosphere coupling and tree functions. *Agricultural and Forest Meteorology*, **97**, 171–185.

Jarvis, P.G. (1976) The Interpretation of the Variations in Leaf Water Potential and Stomatal Conductance Found in Canopies in the Field. *Philosophical Transactions of the Royal Society B: Biological Sciences*, **273**, 593–610.

Massonnet, C., Regnard, J.L., Lauri, P.E., Costes, E. & Sinoquet, H. (2008) Contributions of foliage distribution and leaf functions to light interception, transpiration and photosynthetic capacities in two apple cultivars at branch and tree scales. *Tree Physiology*, **28**, 665–678.

Moon, P. & Spencer, D.E. (1942) Illumination from a non-uniform sky. *Transactions of the Illumination Engineering Society*, **37**, 707–712.

Nobel, P.S. (1999) *Physicochemical & Environmental Plant Physiology*. Academic Press, New York.

Pincebourde, S. & Casas, J. (2006) Leaf miner-induced changes in leaf transmittance cause variations in insect respiration rates. *Journal of Insect Physiology*, **52**, 194–201.

Pincebourde, S., Sinoquet, H., Combes, D. & Casas, J. (2007) Regional climate modulates the canopy mosaic of favourable and risky microclimates for insects. *Journal of Animal Ecology*, **76**, 424–438.

Saudreau, M., Pincebourde, S., Dassot, M., Adam, B., Loxdale, H.D. & Biron, D.G. (2013) On the canopy structure manipulation to buffer climate change effects on insect herbivore development. *Trees*, **27**, 239–248.

Sinoquet, H., Le Roux, X., Adam, B., Ameglio, T. & Daudet, F. a. (2001) RATP: a model for simulating the spatial distribution of radiation absorption, transpiration and photosynthesis within canopies: application to an isolated tree crown. *Plant, Cell and Environment*, **24**, 395–406.


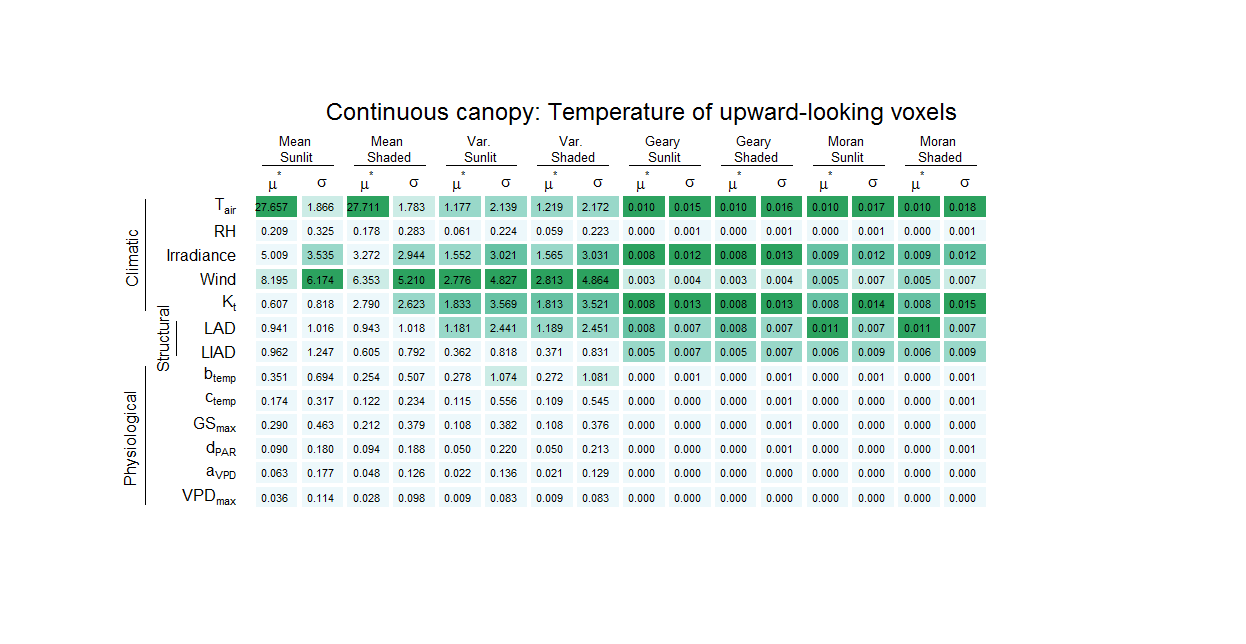

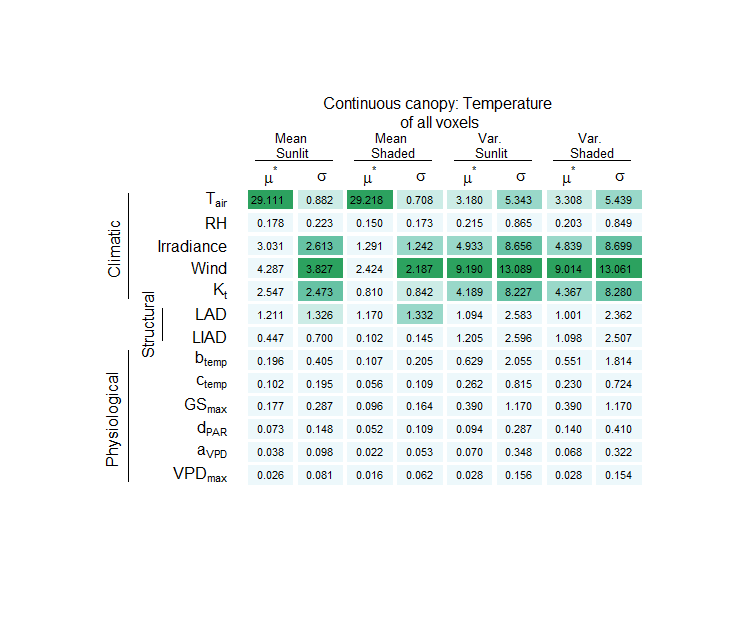
**S2. Tables of the Morris analysis for the simulated continuous canopy, with values of σ and μ* for top viewed voxels versus all voxels. Cells are color-coded within columns by their relative values (darker green = larger number).**


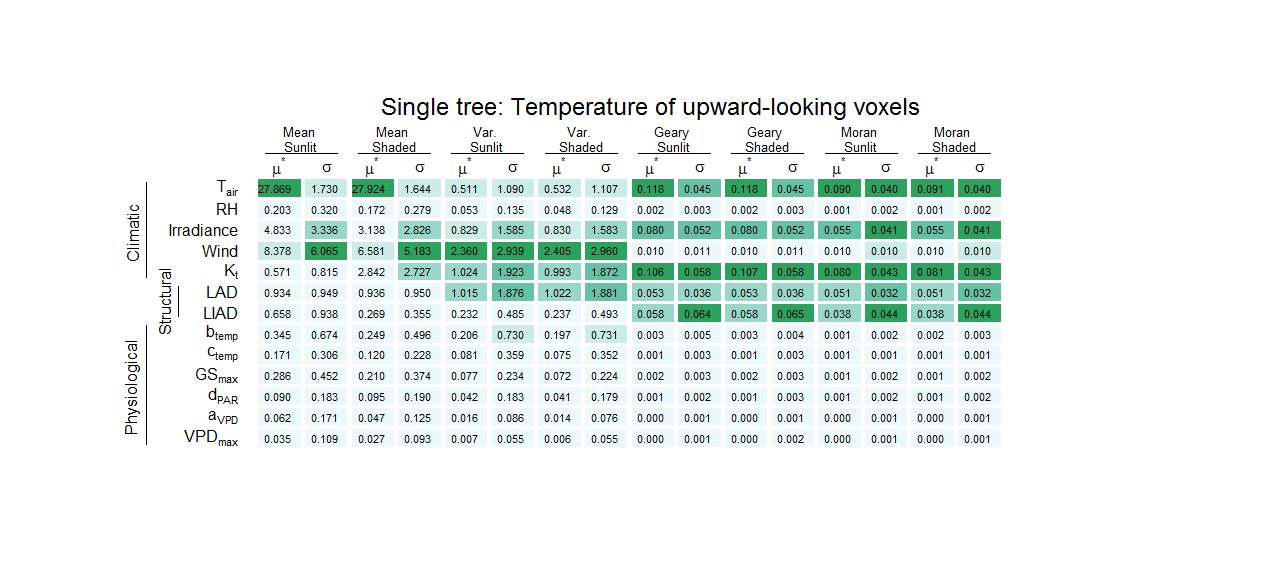

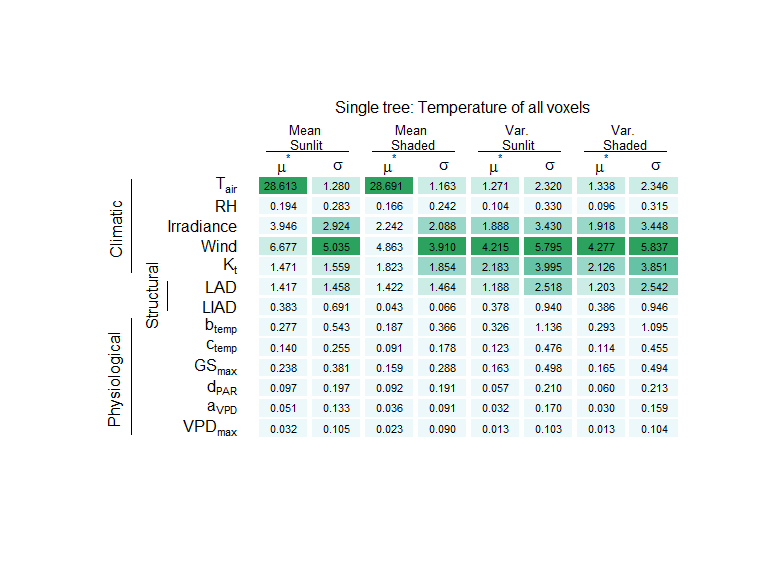
**S3. Tables of the Morris analysis for an isolated tree, with values of σ and μ* for top viewed voxels versus all voxels. Cells are color-coded within columns by their relative values (darker green = larger number).**

**S4. Mean temperature of sunlit portions of voxels throughout the continuous canopy (3D) as a function of the mean of just top voxels (i.e., that could be seen from a drone or a satellite), color coded by selected parameters.**


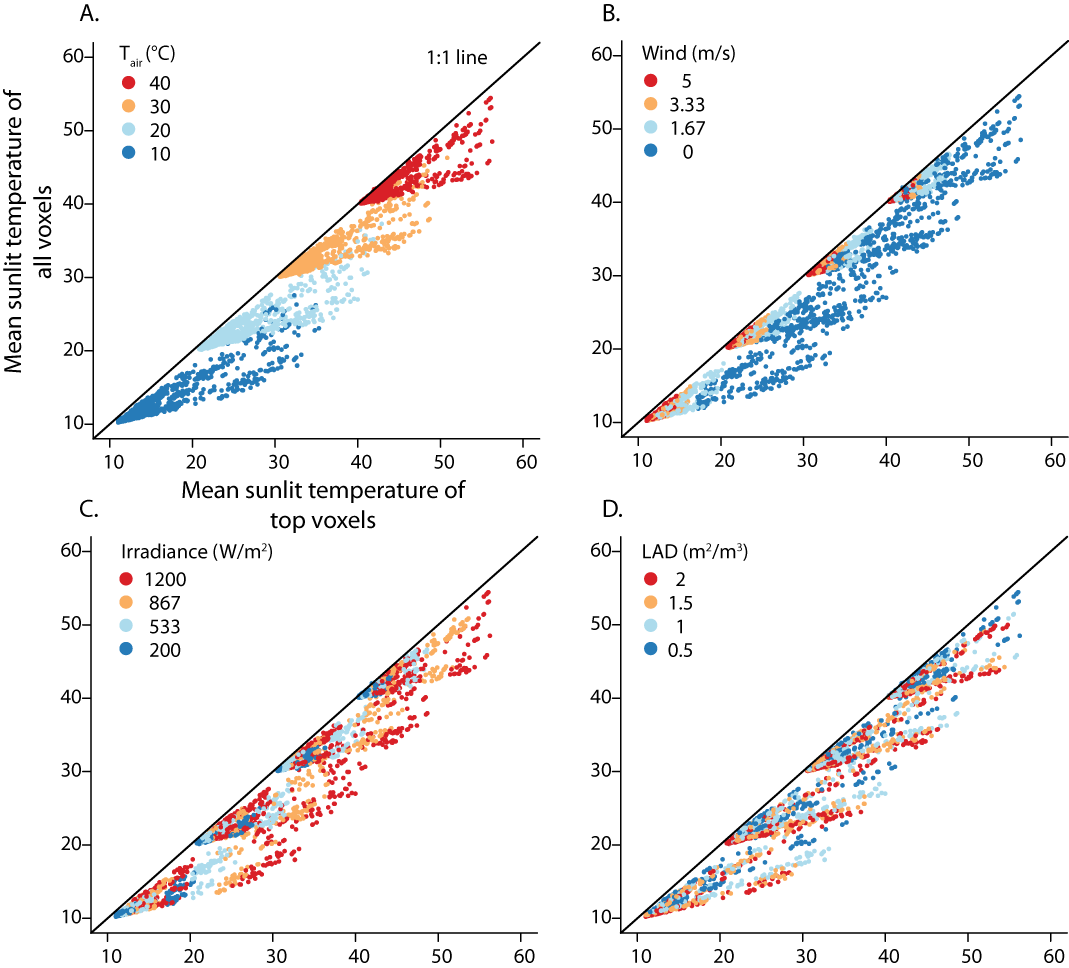


**S5. Mean temperature of shaded portions of voxels throughout the continuous canopy (3D) as a function of the mean of just top voxels (i.e., that could be seen from a drone or a satellite), color coded by selected parameters.


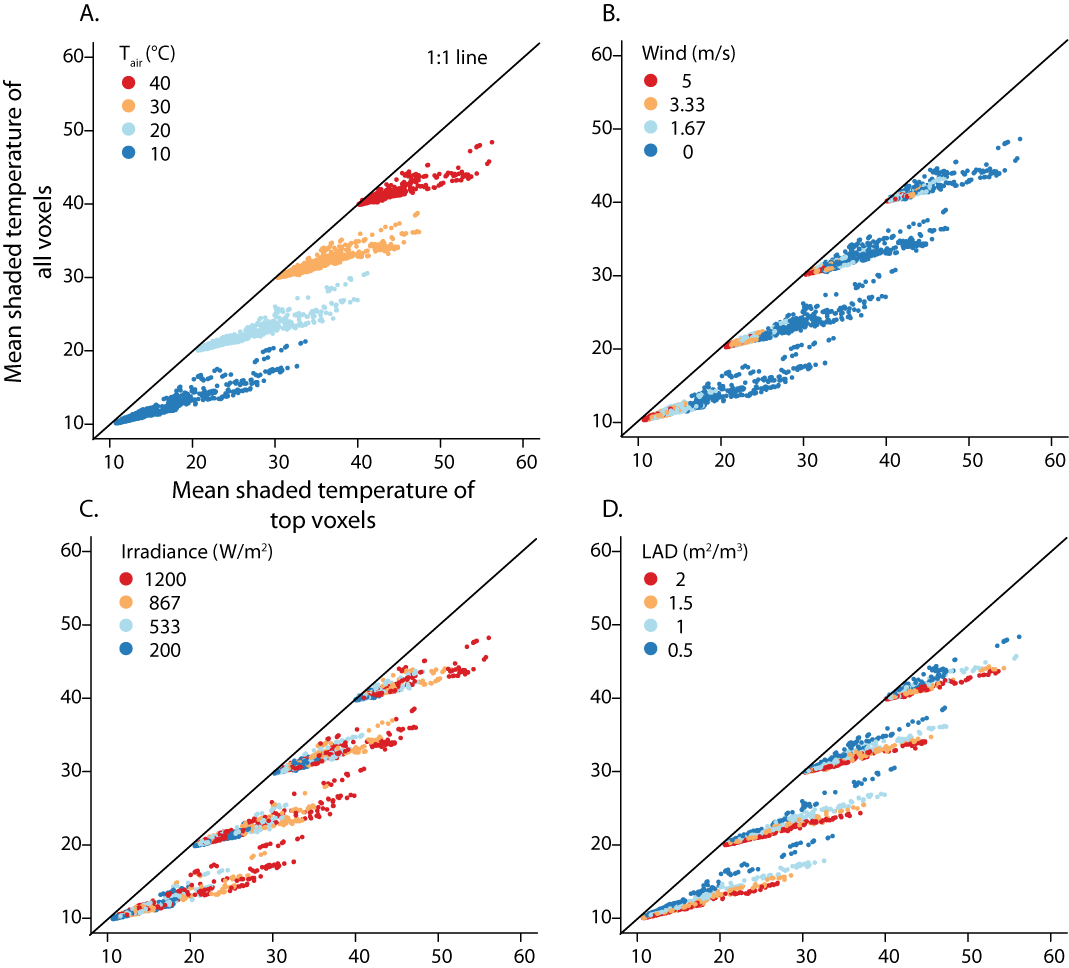
**

**S6. Variance in temperature of sunlit portions of voxels throughout the continuous canopy (3D) as a function of the variance of just top voxels (i.e., that could be seen from a drone or a satellite), color coded by selected parameters.


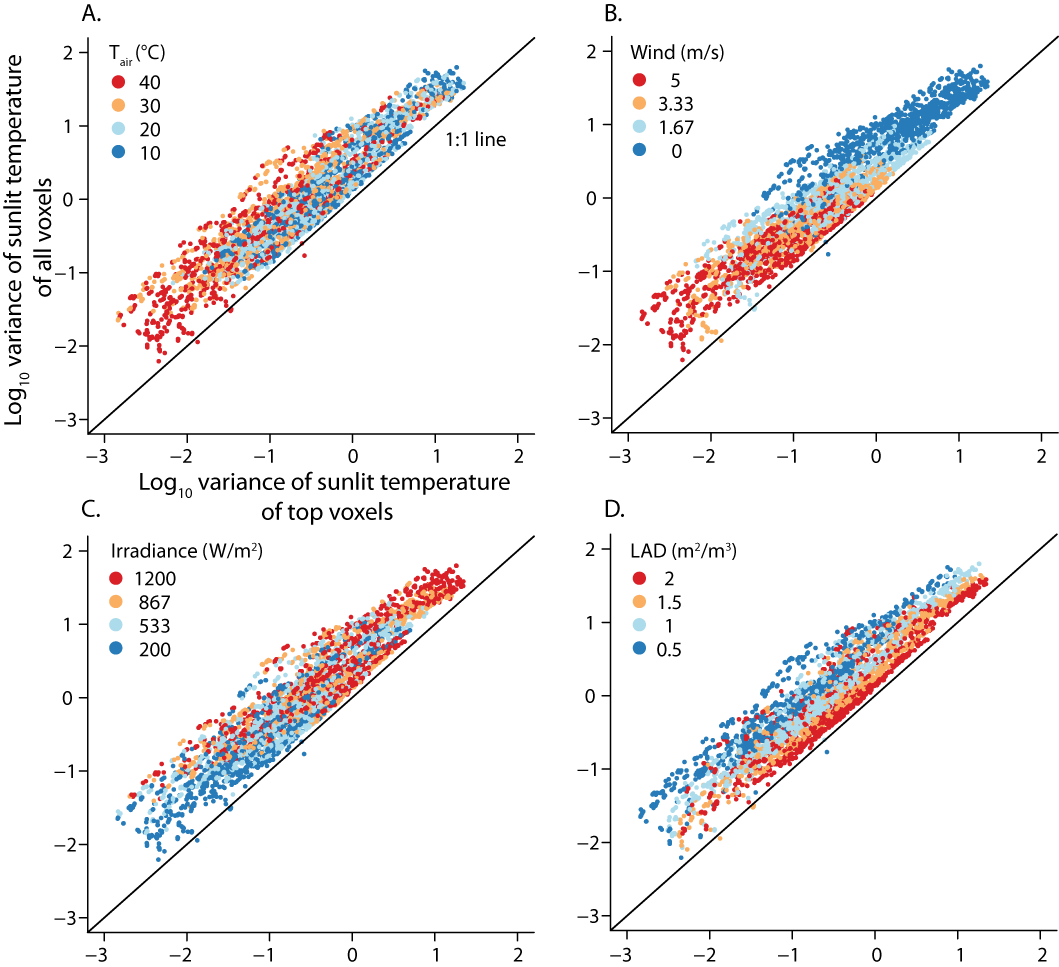
**

**S7. Variance in temperature of shaded portions of voxels throughout the continuous canopy (3D) as a function of the variance of just top voxels (i.e., that could be seen from a drone or a satellite), color coded by selected parameters.


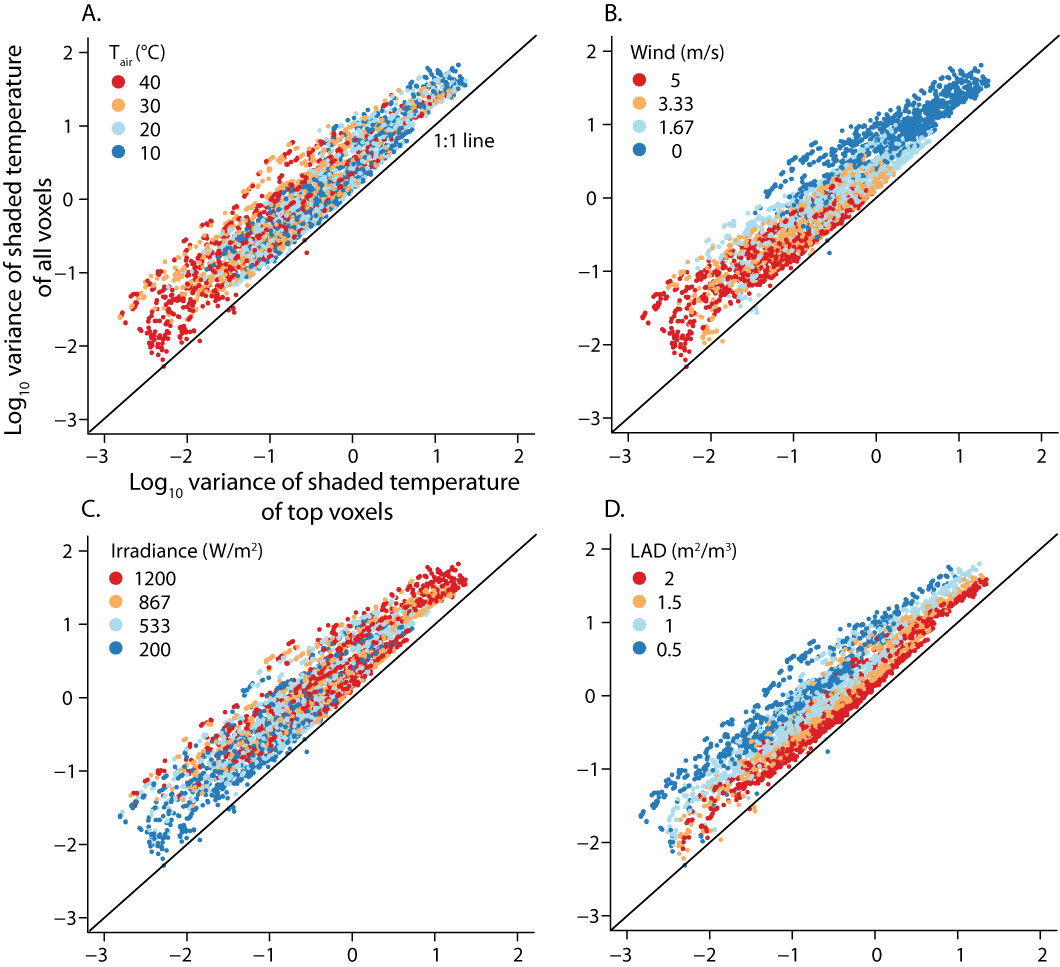
**
